# Ecological Adaptation and Succession of Human Fecal Microbial Communities in an Automated *In Vitro* Fermentation System

**DOI:** 10.1128/mSystems.00232-21

**Published:** 2021-07-27

**Authors:** Thiyagarajan Gnanasekaran, Juliana Assis Geraldo, David Wilczek Ahrenkiel, Camila Alvarez-Silva, Carmen Saenz, Adnan Khan, Obaida Hanteer, Vithiagaran Gunalan, Kajetan Trost, Thomas Moritz, Manimozhiyan Arumugam

**Affiliations:** a Novo Nordisk Foundation Center for Basic Metabolic Research, Faculty of Health and Medical Sciences, University of Copenhagen, Copenhagen, Denmark; University of Trento

**Keywords:** fecal microbiota, *in vitro* fermentor

## Abstract

Longitudinal studies of gut microbiota following specific interventions are vital for understanding how they influence host health. However, robust longitudinal sampling of gut microbiota is a major challenge, which can be addressed using *in vitro* fermentors hosting complex microbial communities. Here, by employing 16S rRNA gene amplicon sequencing, we investigated the adaptation and succession of human fecal microbial communities in an automated multistage fermentor. We performed two independent experiments using different human donor fecal samples, one configured with two units of three colon compartments each studied for 22 days and another with one unit of two colon compartments studied for 31 days. The fermentor maintained a trend of increasing microbial alpha diversity along colon compartments. Within each experiment, microbial compositions followed compartment-specific trajectories and reached independent stable configurations. While compositions were highly similar between replicate units, they were clearly separated between different experiments, showing that they maintained the individuality of fecal inoculum rather than converging on a fermentor-specific composition. While some fecal amplicon sequence variants (ASVs) were undetected in the fermentor, many ASVs undetected in the fecal samples flourished *in vitro*. These bloomer ASVs accounted for significant proportions of the population and included prominent health-associated microbes such as Bacteroides fragilis and Akkermansia muciniphila. Turnover in community compositions is likely explained by feed composition and pH, suggesting that these communities can be easily modulated. Our results suggest that *in vitro* fermentors are promising tools to study complex microbial communities harboring important members of human gut microbiota.

**IMPORTANCE** *In vitro* fermentors that can host complex gut microbial communities are promising tools to investigate the dynamics of human gut microbiota. In this work, using an automated *in vitro* gut fermentor consisting of different colon compartments, we investigated the adaptation dynamics of two different human fecal microbial communities over 22 and 31 days. By observing the temporal trends of different community members, we found that many dominant members of the fecal microbiota failed to maintain their dominance *in vitro*, and some of the low-abundance microbes undetected in the fecal microbiota successfully grew in the *in vitro* communities. Microbiome compositional changes and blooming could largely be explained by feed composition and pH, suggesting that these communities can be modulated to desired compositions via optimizing culture conditions. Thus, our results open up the possibility of modulating *in vitro* microbial communities to predefined compositions by optimizing feed composition and culture conditions.

## INTRODUCTION

The gut microbiota is a vibrant and diverse community consisting of more than 1,000 bacterial and archaeal species that play a vital role in maintaining host health and well-being (1, 2). Various factors, such as diet, drugs, lifestyle, and host immune system, affect the dynamics of the gut microbiota, and this exerts a significant influence on host metabolic homeostasis (3). Hence, understanding the complex dynamics of the gut microbiota is crucial, especially via longitudinal gut microbiota studies.

Longitudinal gut microbiota studies help not only in decoding the changes in microbial composition and function over time but also in reliably associating these changes with host health and disease conditions. To date, a number of longitudinal studies have been carried out to understand the dynamics of gut microbiota under both healthy and disease conditions (4–6). However, such studies have several drawbacks that limit our interpretations of longitudinal dynamics of the gut microbiota. First, most studies use the fecal microbiota as a representative of the gut microbiota, while the composition and gene expression patterns of the fecal microbiota might be quite different from that of the gut microbiota. Second, consistent and frequent (e.g., daily) sampling of fecal material can be difficult. Third, it is difficult to separate longitudinal gut microbial dynamics from effects of dietary changes and the host immune system. Finally, investigating the effects of certain interventions on human gut microbiota might raise ethical concerns. For example, manipulating the microbiota of an individual (e.g., introduction of a probiotic strain or fecal microbial transplantation) could affect microbiotas of cohabiting individuals or family members who had not consented to that intervention, and evaluating interventions that significantly affect the gut microbiota in humans could irreversibly damage host-microbiome homeostasis (7). *In vitro* gut models can complement *in vivo* longitudinal studies to assess the dynamics of gut microbiota. They can simulate gastrointestinal conditions in a precisely controlled manner, allow sampling of active microbial communities in culture, allow consistent and frequent sampling, allow modulation of diet on a flexible schedule, are free from host influence, support culturing and characterization of engineered synthetic gut microbial communities, and do not require ethical approvals (8, 9).

Currently, most available gut models are based on (i) static batch fermentation systems involving small reactors or culture tubes, (ii) semicontinuous fermentation systems involving single fermentors (10–13), or (iii) multistage fermentation continuous systems that involve two or more fermentors connected in series, such as the Simulator of the Human Intestinal Microbial Ecosystem (SHIME) (14), TIM gastrointestinal model (15), PolyFermS model (16), SIMulator of the GastroIntestinal tract (SIMGI) (17) model, Lacroix model, EnteroMix, and CoMiniGut (18–20). Among the available gut models, SHIME is widely used due to (i) its automation, (ii) the possibility of sampling larger volumes more frequently (although this benefit comes with a requirement for large quantity of feed medium), (iii) the possibility of tightly controlling pH, temperature, and flow rate between the colon regions, (iv) the flexibility to configure multiple parallel units and different compartments, and (v) the possibility of performing longitudinal sampling in consecutive colon regions (14, 21, 22). While a majority of studies used SHIME to test the effects of drugs, xenobiotics, and supplements on *in vitro* microbial communities (21, 23–26), it can also be used to study microbial ecology (27, 28).

Recent developments in mass spectrometry and high-throughput sequencing technologies are enabling the identification of novel microbial-derived metabolites in the gut and their respective biosynthetic pathways at a much higher pace than before (29–31). Though many metabolites have been discovered from *in vivo* studies, this discovery process can be accelerated by employing *in vitro* gut models. While metabolites identified from fecal or gut samples could come from host, diet, or microbiota, the major advantage of *in vitro* gut models in metabolite discovery is that any identified nonmedium metabolite can be clearly attributed to the microbiota.

In this study, we used 16S rRNA gene amplicon sequencing to investigate the adaptation and succession of two different fecal microbial communities over a period of 22 and 31 days using an automated *in vitro* fermentation system.

## RESULTS AND DISCUSSION

Using the SHIME system, we ran two independent experiments (experiment 1 [Exp1] and experiment 2 [Exp2]) with fecal samples obtained from two anonymous human donors (donor 1 and donor 2, respectively). We performed Exp1 with two identical units consisting of ascending (AC), transverse (TC), and descending (DC) colon compartments over 22 days and Exp2 with one unit consisting of AC and DC compartments over 31 days (Fig. S1). We characterized the microbiome from these experiments using 16S rRNA gene V4 variable region amplicon sequencing (see Materials and Methods). We generated 3,031,524 high-quality paired-end reads (median, 20,760 per sample) from 3 fecal inoculum replicates and 143 *in vitro* samples in Exp1 and 8,117,537 paired-end reads (median, 231,733 per sample) from one fecal inoculum sample and 32 *in vitro* samples in Exp2. Due to the major difference in sequencing depth between the two experiments, we rarefied all samples to 20,000 high-quality paired-end reads (see Materials and Methods). After merging the rarefied read pairs and removing chimeric amplicons, we obtained 2,279,301 (and 417,882) high-quality amplicon sequences from Exp1 (and Exp2) with 15,682 (and 12,440) median amplicons per sample. From this, we derived 424 amplicon sequencing variants (ASVs) across the two experiments using the DADA2 pipeline (32). In Exp1, we confirmed that the microbiome communities in the two identical units were quite similar in both alpha diversity (ASV richness and ASV Shannon index) and beta diversity (Jensen-Shannon distance based on ASV relative abundances) by comparing corresponding samples (Fig. S2 and S3A). Additionally, using samples collected as technical triplicates on days 2, 7, 12, 17, and 22, we verified the reproducibility of our sample processing and analytical pipeline, measured by alpha diversity (Fig. S3B and S3C) and beta diversity (Fig. S3D). For these time points in Exp1, we consistently chose the second replicate for all further analyses.

10.1128/mSystems.00232-21.1 FIG S1 Schematic representation of the SHIME setups used in Exp1 (A) and Exp2 (B). ST, stomach; SI, small intestine; ST+SI, combined stomach and small intestine; AC, ascending colon; TC, transverse colon; DC, descending colon; SF, SHIME feed; PJ, pancreatic juice; acid, 0.5 M HCl; base, 0.5 M NaOH. Download FIG S1, PDF file, 0.2 MB. Copyright © 2021 Gnanasekaran et al. 2021 Gnanasekaran et al. https://creativecommons.org/licenses/by/4.0/ This content is distributed under the terms of the Creative Commons Attribution 4.0 International license.

10.1128/mSystems.00232-21.2 FIG S2 Microbial ASV richness (top row) and Shannon diversity (bottom row) observed in the AC, TC, and DC compartments of Exp1 (left) and AC and DC compartments of Exp2 (right) over 22 and 31 days, respectively. F, fecal sample. Download FIG S2, PDF file, 0.05 MB. Copyright © 2021 Gnanasekaran et al. 2021 Gnanasekaran et al. https://creativecommons.org/licenses/by/4.0/ This content is distributed under the terms of the Creative Commons Attribution 4.0 International license.

10.1128/mSystems.00232-21.3 FIG S3 Quality control analysis in Exp1 using matched samples from parallel units of SHIME and triplicate samples on select days. (A) Pairwise comparison between matched samples from parallel units of SHIME in Exp1 using Jensen-Shannon distance. (B to D) Comparison of alpha and beta diversity in technical triplicate samples on select days from Exp1. ASV richness (B), ASV Shannon diversity (C), and beta diversity using Jensen-Shannon distance (D) obtained for days 2, 7, 12, 17, and 22 from all the three colon compartments. Download FIG S3, PDF file, 0.3 MB. Copyright © 2021 Gnanasekaran et al. 2021 Gnanasekaran et al. https://creativecommons.org/licenses/by/4.0/ This content is distributed under the terms of the Creative Commons Attribution 4.0 International license.

### Different colon compartments reach individual stable microbiome compositions.

We detected 117 ASVs in donor 1 fecal inoculum, while in the AC, TC, and DC compartments from Exp1, we detected on average 39, 71, and 82 ASVs, respectively, across the study period. In the case of Exp2, we detected 79 ASVs in donor 2 fecal inoculum, while in the AC and DC compartments, we detected on average 36 and 66 ASVs, respectively. This suggests that some ASVs in the fecal samples from both experiments either were permanently lost or went below our detection threshold. To identify our detection threshold, we performed cell counting on the fecal sample in Exp2 and estimated a cell density of 9.3 × 10^10^ cells/g. At a rarefied read depth of 15,000, we estimated our detection threshold to be 6.2 × 10^6^ cells/g of feces. In addition, the biases inherent to DNA extraction and 16S rRNA gene amplicon sequencing library preparation could affect the detection threshold (33, 34).

In Exp1, after an irregular trend in the first 2 days, the ASV richness declined in all compartments over the week from D5 to D12 (Fig. S2, top left). However, from D12 onward, ASV richness reached different stable values in all three compartments. On the other hand, Shannon diversity of all the compartments in Exp1 showed a declining trend until D12 starting from D2 and thereafter reached stable values only in DC but exhibited an increasing trend in AC and TC (Fig. S2, bottom left). In Exp2, after a declining trend of ASV richness for the first 7 days, it reached stable values in both the compartments (Fig. S2, top right). However, the Shannon diversity of both compartments showed increasing trends until D23, starting from D9 in AC and from D3 in DC. Then, starting from D25, both compartments exhibited instability until the end of the experiment (Fig. S2, bottom right). The colon compartments maintained a trend of increasing alpha diversity from AC to TC to DC compartments for Exp1 and from AC to DC for Exp2 (Fig. S2). This pattern of increasing alpha diversity along AC, TC, and DC compartments is in accordance with an earlier *in vitro* study that employed SHIME (22).

**FIG 1:**
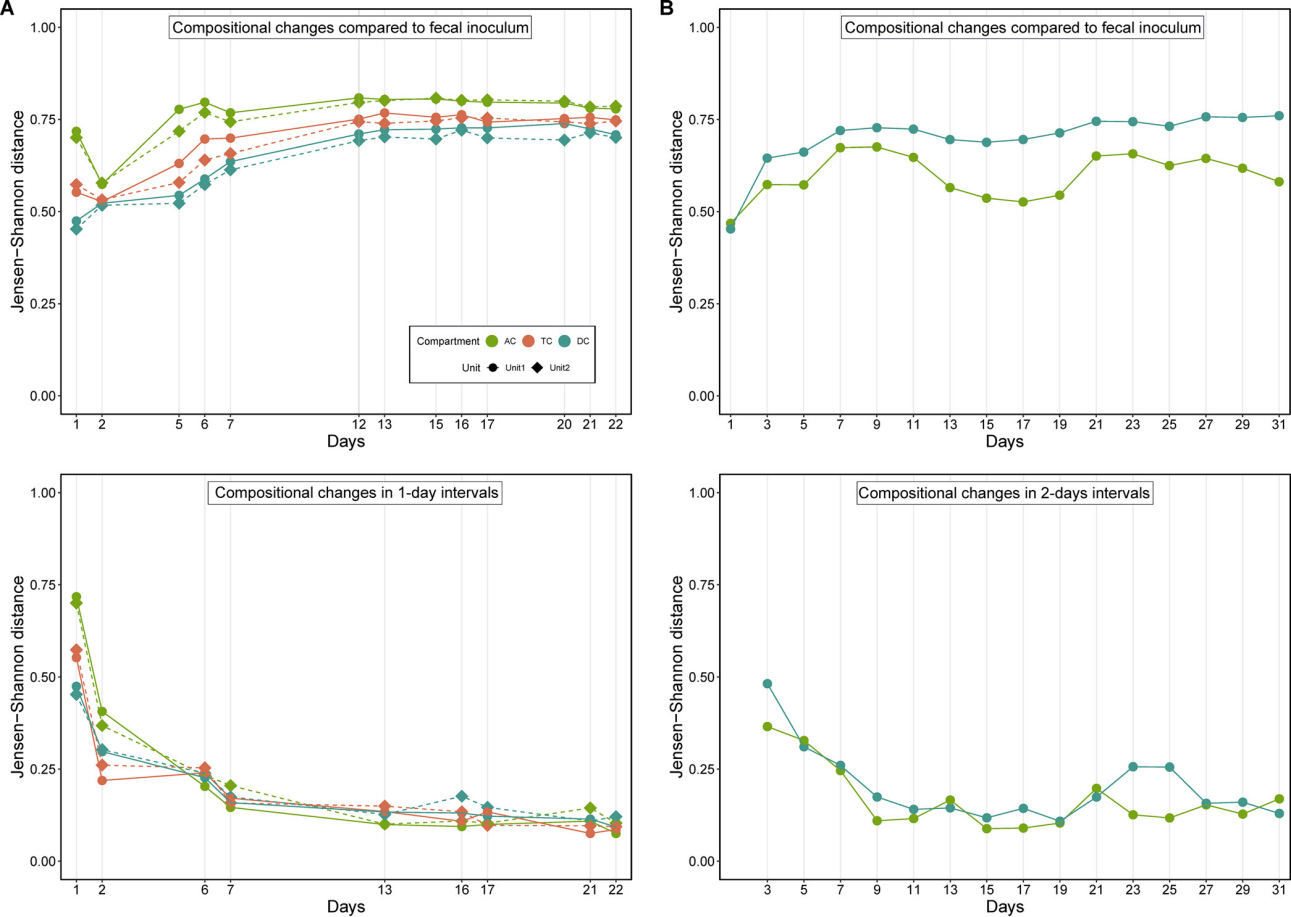
Microbiome compositions stabilize in different compartments of the *in vitro* fermentor. (A) Microbial beta diversity of the colon compartments compared to the fecal inoculum (top) and during 1-day intervals (bottom) in Exp1. (B) Microbial beta diversity of the colon compartments compared to the fecal inoculum (top) and during 2-day intervals (bottom) in Exp2. Beta diversity was calculated using Jensen-Shannon distance.

Next, we investigated community stability in the different compartments in both experiments. For this purpose, we estimated the beta diversity of *in vitro* samples compared to fecal inoculum and observed that *in vitro* communities stabilized around D12 in Exp1 (Fig. 1A, top). Since beta diversity measures are thought to saturate at higher magnitude (35), we also estimated community compositional changes at 1-day intervals, which further confirmed that the microbiome communities stabilized at this time point (Fig. 1A, bottom). Similar analysis showed that microbiome compositions also stabilized in Exp2 around D13 (Fig. 1B). Comparing the microbiome compositions of fecal inoculum to samples from all colon compartments, we found that the DC microbiome was closest to the fecal microbiome throughout the experiment, followed by TC and AC microbiomes, in Exp1 (Fig. 1A), whereas the AC microbiome was closest to the fecal inoculum in Exp2 (Fig. 1B). In addition, the highest separation in Exp1 was observed between AC and DC, followed by AC/TC and TC/DC separations (Fig. S4).

10.1128/mSystems.00232-21.4 FIG S4 Microbiome compositional differences between AC-TC, TC-DC, and AC-DC compartments in Exp1 using Jensen-Shannon distance as the beta diversity measure. Download FIG S4, PDF file, 0.04 MB. Copyright © 2021 Gnanasekaran et al. 2021 Gnanasekaran et al. https://creativecommons.org/licenses/by/4.0/ This content is distributed under the terms of the Creative Commons Attribution 4.0 International license.

**FIG 2:**
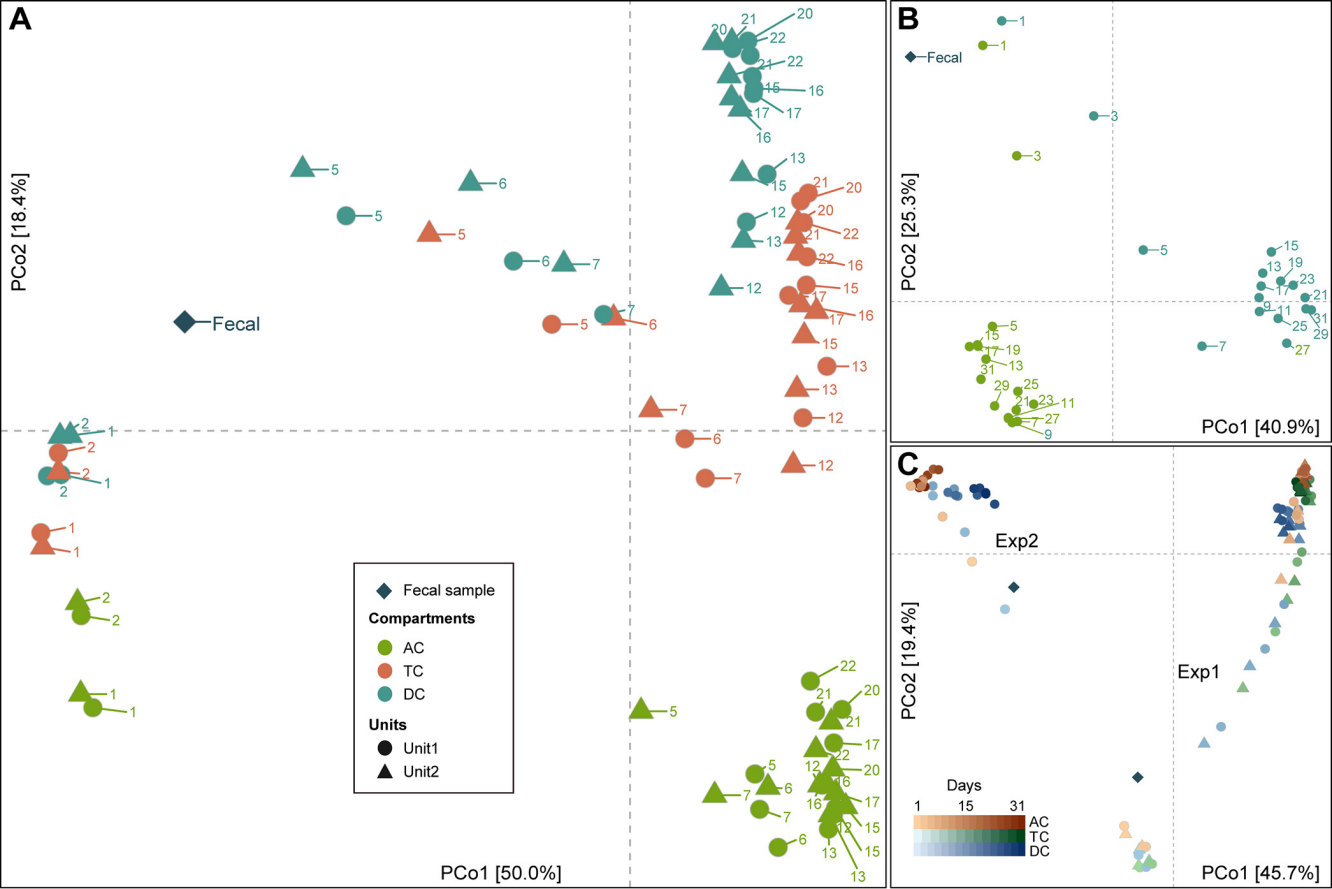
Projection of the first two principal coordinates in the microbiome composition in the two experiments. Microbiome compositions in the parallel units in Exp1 exhibit similar trajectories over time (A). Different colon compartments follow different trajectories in Exp1 (A) and Exp2 (B). Microbiome compositions in Exp1 and Exp2 also follow distinct trajectories in a combined analysis (C). Samples are labeled with the day of the experiment in panels A and B and colored with a gradient corresponding to the day of the experiment in panel C. Beta diversity was calculated using Jensen-Shannon distance.

We visualized the longitudinal compositional changes in both experiments by performing principal-coordinate analysis (PCoA) on the beta diversity and visualizing the first two coordinates. In Exp1, microbiome compositions of different compartments changed dramatically over the first few days and diverged from each other (Fig. 2A). They maintained these diverged compositions, followed different trajectories, and later stabilized at their own individual steady-state compositions. Similarly, in Exp2, AC and DC microbiomes followed different trajectories and stabilized to steady-state compositions after D9 (Fig. 2B). This divergence observed between the compartments in both experiments could be due to the differences in pH, availability of carbohydrates, or bile salt concentration across these compartments (22). A combined PCoA showed that Exp1 and Exp2 microbiomes followed independent trajectories and reached different stable compositions (Fig. 2C), demonstrating that microbial compositions of colonic compartments and their trajectories depend on the composition of the fecal inoculum, thus maintaining individuality, and more importantly that they do not converge toward a common composition inherent to the batch fermentation system (36).

Previous *in vitro* studies employed short-chain fatty acid (SCFA) concentrations as a proxy to determine microbial community stability (37–39). SCFAs are predominantly produced by a limited number of bacteria (40), and there is compelling evidence from both *in vitro* and *in vivo* studies that the SCFA concentrations might not reflect the community structure (41–43). Also, cross-feeding on the SCFAs by other members of a community is inevitable in both *in vivo* and *in vitro* settings, which could introduce bias in the quantified SCFA levels (44, 45). Therefore, our use of microbiome beta diversity from amplicon sequencing assesses community stability more accurately.

In both experiments, microbial communities in the DC compartment exhibited the highest richness (Fig. S2) and harbored a different composition (Fig. 2) compared to other compartments. While this has been observed before (22), previous studies have not investigated whether this (i) was merely due to the cumulative wash-through of the microbes from the preceding compartments combined with new bacterial growth or (ii) stemmed from the establishment of a different actively growing richer community due to the differences in conditions (e.g., pH, availability of carbohydrates, or bile salt concentrations) between DC and other compartments. As relative abundance cannot reveal whether ASVs expanded in DC, we estimated ASV absolute abundances for Exp2 by performing cell counts on all samples (Fig. S5A) and compared them between AC and DC in all time points (Fig. S5B). While several ASVs detected in AC were not detected in DC (11.1% to 32.5% across all time points) (Fig. S5C), most were detected in DC with an abundance difference within an order of magnitude. This suggests that periodic flow from AC to DC could play a major role in shaping DC microbial composition. However, several ASVs increased in abundance from AC to DC while several others decreased, sometimes by more than an order of magnitude (Fig. S5B). Additionally, 51.5% to 61.5% ASVs detected in DC were not detected in AC (Fig. S5C), several reaching an abundance of >10^7^ cells per ml (Fig. S5B), suggesting that DC conditions were less restrictive. As specific examples, Akkermansia muciniphila [4] (numbers in brackets are ASV IDs) thrived well in DC but was not detectable in AC; *Lachnoclostridium* [5] thrived in higher abundance in DC than AC; and ASVs such as Prevotella copri [18], *Prevotella* [42], and *Prevotellaceae* [28] thrived in higher abundance in AC than DC (Fig. S5D). These observations support the hypothesis that DC harbors a richer actively growing community. Due to the interconnected nature of compartments, we cannot rule out flow of microbial strains from preceding reactors to DC contributing to the increased richness and influencing the composition in DC. Nevertheless, our results imply that the conditions in DC play a pivotal role in establishment and maintenance of an active community with higher richness.

10.1128/mSystems.00232-21.5 FIG S5 Tracking ASV composition dynamics in AC and DC compartments from Exp2 over time. (A) Total cell count measurements of bacterial pellets harvested from D1 to D31. Since the measurements included high noise at early time points, we approximated the cell counts using a longitudinal trend (red), which was used to convert relative abundance to absolute abundance in Fig. 4B and Fig. S5B. (B) Comparison of absolute abundances of ASVs in AC and DC compartments. Each panel represents a different day. Lines connect abundances of the same ASV and are colored by class annotation. (C) Number of ASVs unique to or shared between AC and DC compartments at different time points. (D) Longitudinal changes in the absolute abundances of different ASVs observed for AC and DC compartments in Exp2. Download FIG S5, PDF file, 2.1 MB. Copyright © 2021 Gnanasekaran et al. 2021 Gnanasekaran et al. https://creativecommons.org/licenses/by/4.0/ This content is distributed under the terms of the Creative Commons Attribution 4.0 International license.

### Different dynamics exhibited by microbes during the adaptation and succession.

**FIG 3:**
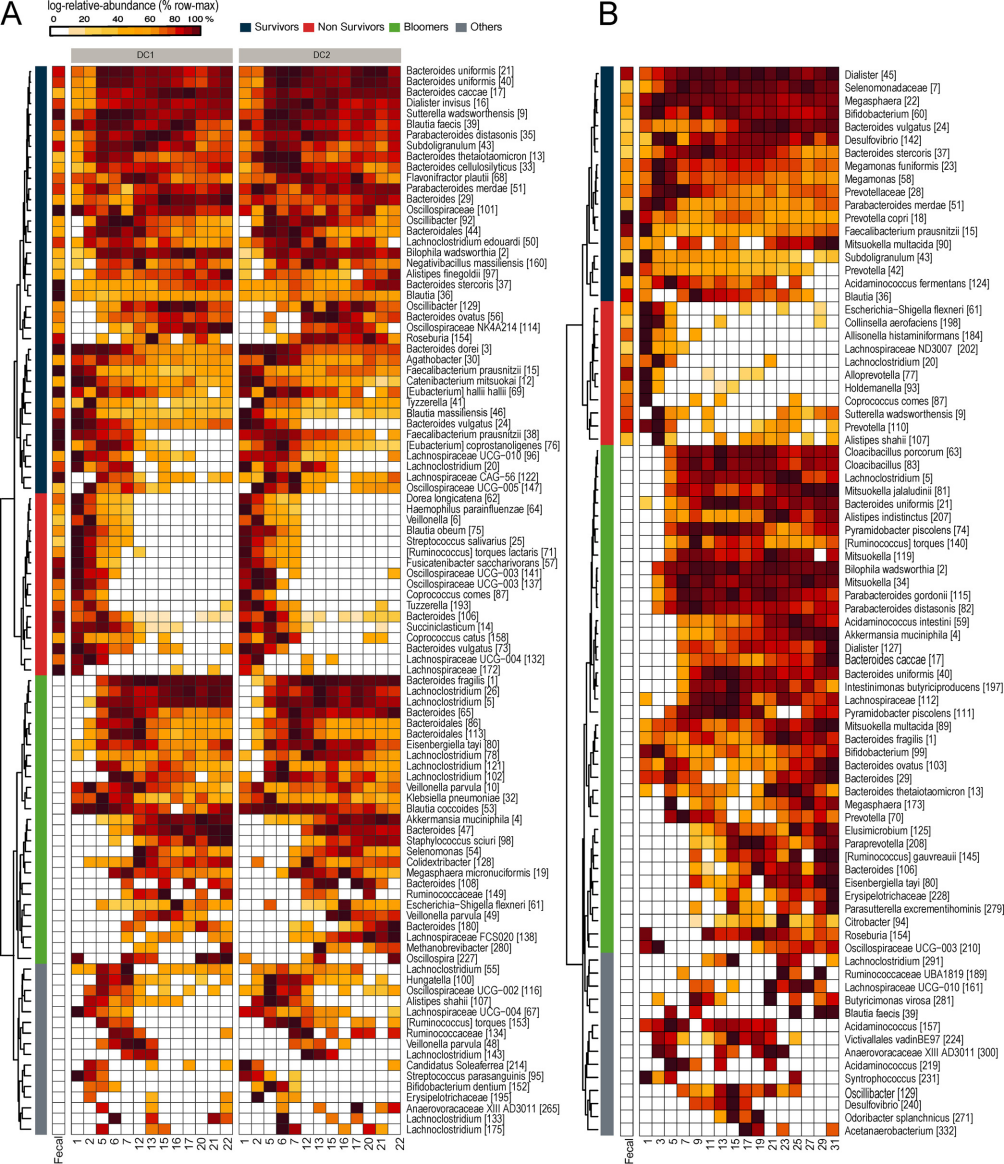
Relative abundance profiles of prominent ASVs in descending colon (DC) compartments from Exp1 (A) and Exp2 (B) highlight three different trends based on hierarchical clustering – survivors, nonsurvivors, and bloomers. Only prominent ASVs detected in at least 3 and 4 samples in Exp1 and Exp2, respectively, are shown here. Relative abundance profiles of all ASVs are shown in Fig. S6. Each row is scaled between 0% to 100% of the maximum log relative abundance of the given ASV.

To gain insights into how microbes adapted to the *in vitro* fermentor, we analyzed longitudinal relative abundance profiles of ASVs that were detected at at least three time points from each DC compartment (prominent ASVs). Based on hierarchical clustering of their profiles, we identified three major trends: survivors, i.e., prominent ASVs detected in the fecal inoculum that successfully survived in the fermentor; nonsurvivors, i.e., ASVs detected in the fecal inoculum that failed to survive in the fermentor or declined in abundance below the detection threshold; and bloomers, i.e., prominent ASVs which were not detected in the fecal inoculum but which bloomed in the fermentor and mostly maintained their population (Fig. 3). In Exp1, survivors, nonsurvivors, and bloomers accounted for 25%, 48%, and 17% of prominent ASVs, while in Exp2 they accounted for 14%, 44%, and 29%. Overall, a high diversity of bacteria representing different genera were present in all three groups, suggesting that none of the groups was dominated by any particular genus.

10.1128/mSystems.00232-21.6 FIG S6 Relative abundance profiles of all the ASVs identified in DC for Exp1 (A) and Exp2 (B) ordered based on hierarchical clustering. See Fig. 5 for an explanation of the heat map. Download FIG S6, PDF file, 0.5 MB. Copyright © 2021 Gnanasekaran et al. 2021 Gnanasekaran et al. https://creativecommons.org/licenses/by/4.0/ This content is distributed under the terms of the Creative Commons Attribution 4.0 International license.

The survival and blooming of ASVs were highly dependent on the fecal inoculum used for seeding. Among the 40 survivors in Exp1 and 18 survivors in Exp2, only six ASVs (Bacteroides stercoris [37], Bacteroides vulgatus [24], Faecalibacterium prausnitzii [15], Parabacteroides merdae [51], *Subdoligranulum* [43] and *Blautia* [36]) were common to both experiments. Similarly, among the 27 bloomers in Exp1 and 38 bloomers in Exp2, only 4 ASVs (Akkermansia muciniphila [4], Bacteroides fragilis [1], *Lachnoclostridium* [5], and Eisenbergiella tayi [80]) were common. Five ASVs belonging to Faecalibacterium prausnitzii were present in considerable abundance in each of the fecal inocula. However, only *F. prausnitzii* [15] survived in both experiments, although with a declining trend. The declining growth of *F. prausnitzii* could be due to the absence of sugars such as lactose in the feed (46) or to its lower fitness in catabolizing mucin present in the feed compared to more efficient mucin catabolizers, such as *A. muciniphila* or some *Bacteroides* spp., that exhibited higher growth (47, 48). In addition, in both experiments, several fecal ASVs could not be detected at any of the time points in the *in vitro* microbiomes. Around 16% of fecal ASVs from both Exp1 (19 of 117) and Exp2 (13 of 79) went undetected in DC compartments (Fig. S6).

During the adaptation and succession, even dominant ASVs in the fecal microbiomes ended up losing their dominance. In the donor 1 fecal microbiome, the five most abundant ASVs together accounted for >50% of relative abundance: Bacteroides dorei [3] (23%), Faecalibacterium prausnitzii [15] (10%), Sutterella wadsworthensis [9] (6%), *Lachnospiraceae* sp. [84] (6%), and *Blautia* sp. [36] (5%). These accounted for only 8% of total relative abundance in the DC compartments after stabilization (3%, 1%, 3%, 0%, and 1% individual average relative abundances, respectively). The same trend was also evident among the 20 most abundant fecal ASVs, which accounted for 80% of relative abundance in the fecal microbiome but a meager 12% in DC microbiomes. In donor 2 fecal microbiome, the five most abundant ASVs accounted for >70% of relative abundance: Prevotella copri [18] (42%), *Prevotella* [42] (17%), *Alloprevotella* [77] (7%), *Prevotellaceae* [139] (4%), and Megamonas funiformis [23] (3%). However, the abundance of these ASVs declined to <10% in the DC compartment.

### Enrichment of rare microbes undetected in the fecal microbiome.

**FIG 4:**
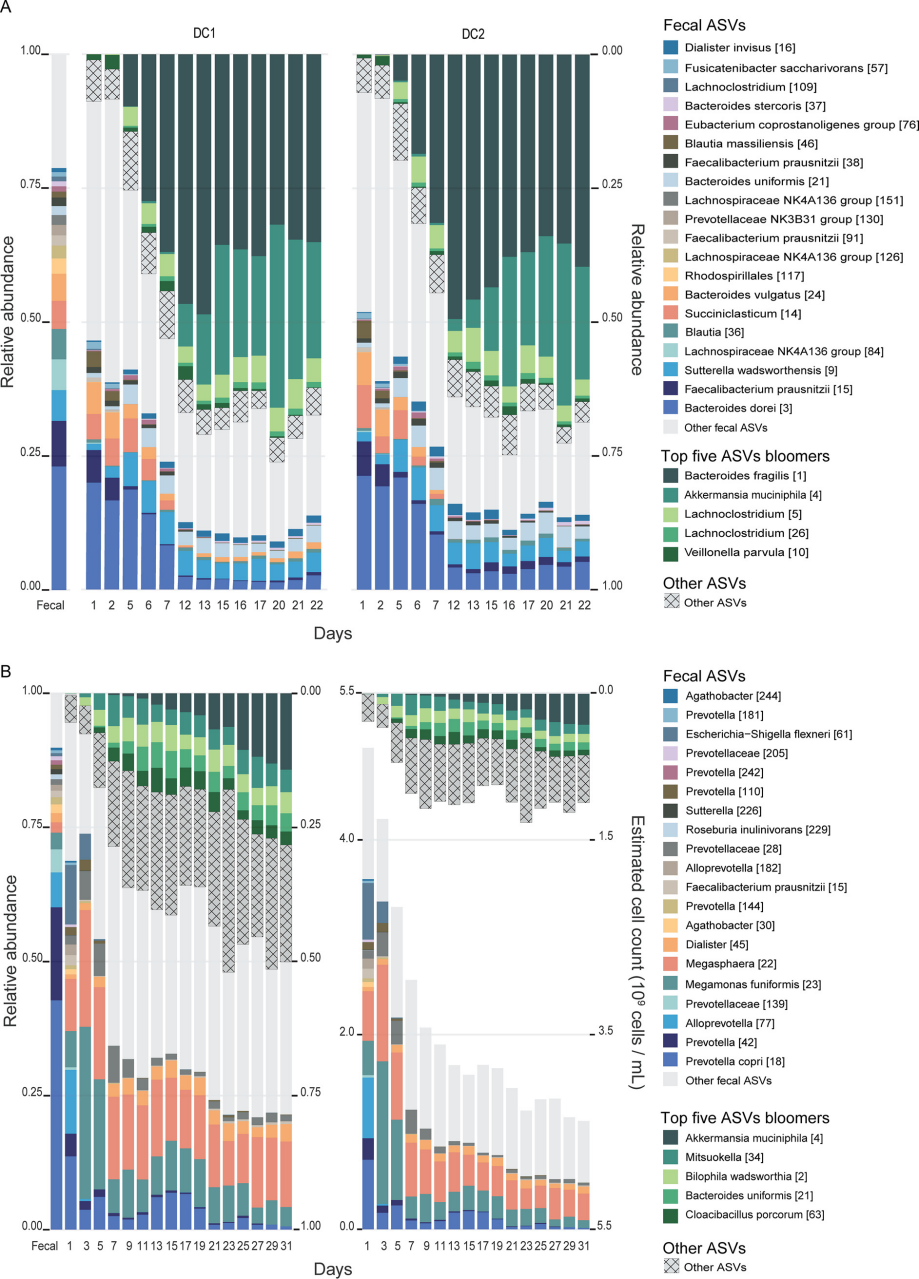
Longitudinal dynamics of the top 5 bloomer ASVs and top 20 fecal ASVs in the DC compartments of Exp1 (A) and Exp2 (B). The 20 most abundant ASVs in the fecal sample (colored boxes at the bottom) accounting for >75% and >85% of relative abundance decreased to <20% and <30% by D12 and D11 in Exp1 and Exp2, respectively. Unshaded gray boxes represent other fecal ASVs. At the same time, the 5 most abundant bloomer ASVs (green boxes at the top) increased from being undetected in the fecal microbiome to >60% by D12 in Exp1 and >30% by D11 in Exp2. Hatched gray boxes represent other bloomer ASVs. Longitudinal dynamics in other compartments are shown in Fig. S7. (A) ASV relative abundances over time in DC compartment of unit 1 (left) and unit 2 (right) from Exp1. (B) ASV relative (left) and absolute (right) abundances over time in DC compartment from Exp2.

Many ASVs that were not detected in the fecal microbiome were consistently detected in the *in vitro* microbiomes in both experiments. These bloomer ASVs were present in the fecal sample but went undetected, as their density was likely below our detection threshold of 6.2 million cells per g of feces. In all three compartments in Exp1, bloomer ASVs accounted for significant proportions of ASV relative abundance (88%, 76%, and 69% in AC, TC, and DC, respectively). For instance, the top 5 bloomer ASVs in the DC compartments (Bacteroides fragilis [1], Akkermansia muciniphila [4], *Lachnoclostridium* [26], *Lachnoclostridium* [5], and Veillonella parvula [10]) constituted >63% of relative abundance after stabilization (Fig. 4A). Similarly, the top 5 bloomer ASVs in the AC compartment constituted >83% and the top 5 bloomer ASVs in the TC compartment constituted >71% of relative abundance (Fig. S7). Compared to Exp1, the bloomer ASVs in Exp2 accounted for lower proportions of ASV relative abundance (18% in AC and 43% in DC). In the same vein, the top 5 bloomer ASVs in DC (*A. muciniphila* [4], *Mitsuokella* [34], Bilophila wadsworthia [2], Bacteroides uniformis [21], and Cloacibacillus porcorum [63]) constituted only 21%, and the top 5 bloomers in AC constituted only 13%.

10.1128/mSystems.00232-21.7 FIG S7 Longitudinal dynamics of top 5 bloomer ASVs and top 20 fecal ASVs observed in AC compartments of Exp1 (A) and Exp2 (B), and the TC compartment of Exp1 (C). The top 5 bloomer ASVs that were not detected in feces but constituted >83% relative abundance of AC in Exp1 and >15% of AC in Exp2 and also >71% of TC in Exp1 after stabilization are shown in shades of green at the top. Hatched gray boxes represent other bloomer ASVs. At the bottom are the relative abundances of the top 20 most abundant ASVs detected in the feces and the shift in their relative abundances in AC and TC compartments over a period of 22 days in Exp1 and 31 days in Exp2. Unshaded gray boxes represent other fecal ASVs. (A) ASV relative abundances over time in the AC compartment of unit 1 (left) and unit 2 (right) from Exp1. (B) ASV relative (left) and absolute (right) abundances over time in AC compartment from Exp2. (C) ASV relative abundances over time in the TC compartment of unit 1 (left) and unit 2 (right) from Exp1. Download FIG S7, PDF file, 1.2 MB. Copyright © 2021 Gnanasekaran et al. 2021 Gnanasekaran et al. https://creativecommons.org/licenses/by/4.0/ This content is distributed under the terms of the Creative Commons Attribution 4.0 International license.

In Exp1, the bloomer B. fragilis [1] was the most abundant ASV after stabilization in AC (60%), TC (50%), and DC (40%) compartments (Fig. 4A; Fig. S7), potentially due to its fitness over other *Bacteroides* species such as *B. dorei* [3], which was the most abundant ASV (23%) in donor 1 fecal microbiome, in utilizing a wide variety of carbon sources present in our feed (49, 50). In Exp2, on the other hand, B. fragilis [1] was not detected in the donor 2 fecal microbiome, and its relative abundance was less than 1% in the *in vitro* samples after stabilization. Instead, B. uniformis [21] bloomed in the DC compartment, albeit to a maximum of 5.1% relative abundance. Donor-dependent trajectories were also observed at other taxonomic levels. For example, the genus *Bacteroides* maintained its dominance in Exp1 (33% in donor 1 fecal microbiome and 30 to 62% in the *in vitro* samples), whereas it maintained a lower abundance in Exp2 (<0.1% in donor 2 fecal microbiome and 3% to 18% in the *in vitro* samples) (Fig. S8). Such a large difference in *Bacteroides* genus abundance despite the same nutrient composition likely derives from the different ecological constraints inherited from the donor fecal microbial communities. This could suggest that the ecological niche for *Bacteroides* in Exp1 had room for a new *Bacteroides* ASV to grow in higher abundance, while the niche in Exp2 did not.

10.1128/mSystems.00232-21.8 FIG S8 Longitudinal changes in relative abundance of the genus *Bacteroides* observed in DC compartments of both Exp1 and Exp2. Download FIG S8, PDF file, 0.02 MB. Copyright © 2021 Gnanasekaran et al. 2021 Gnanasekaran et al. https://creativecommons.org/licenses/by/4.0/ This content is distributed under the terms of the Creative Commons Attribution 4.0 International license.

Another prominent bloomer, *A. muciniphila*, was not detected in the donor 1 or donor 2 fecal samples but bloomed in the TC (14%) and DC (19%) compartments of Exp1 and the DC (8%) compartment of Exp2 after stabilization. Also, in both experiments, *A. muciniphila* continued to increase in relative abundance until the end of the study, suggesting the availability of excess mucin for its catabolism. From relative abundance data, it was not possible to verify whether *A. muciniphila* continued to expand in absolute abundance. When we estimated absolute abundances using cell counts in Exp2 (Fig. S5A), we observed that *A. muciniphila* indeed exhibited a pattern of absolute expansion, while other top ASVs showed a reduction in absolute abundance (Fig. 4B, right). Since *A. muciniphila* is a highly prevalent commensal in healthy humans, its undetected presence in both fecal inocula is not surprising. It normally accounts for less than 1% of total fecal bacteria (51), which likely explains why we did not detect it in the fecal inocula. Its higher growth under the *in vitro* conditions is also not surprising, as the nutrient media have ample amounts of mucin (3 g/liter) (52). However, its higher growth especially in the DC compartments compared to other compartments could be due to the more neutral pH (6.6 to 6.9) conditions (28, 53). This possibility of cultivating and enriching undetectably low-abundance microbes present in feces using *in vitro* fermentors provides a unique opportunity for characterizing rare microbes that cannot be identified using DNA sequencing of the fecal microbiome.

### Coherent microbe-exometabolite groups based on correlation network.

**FIG 5:**
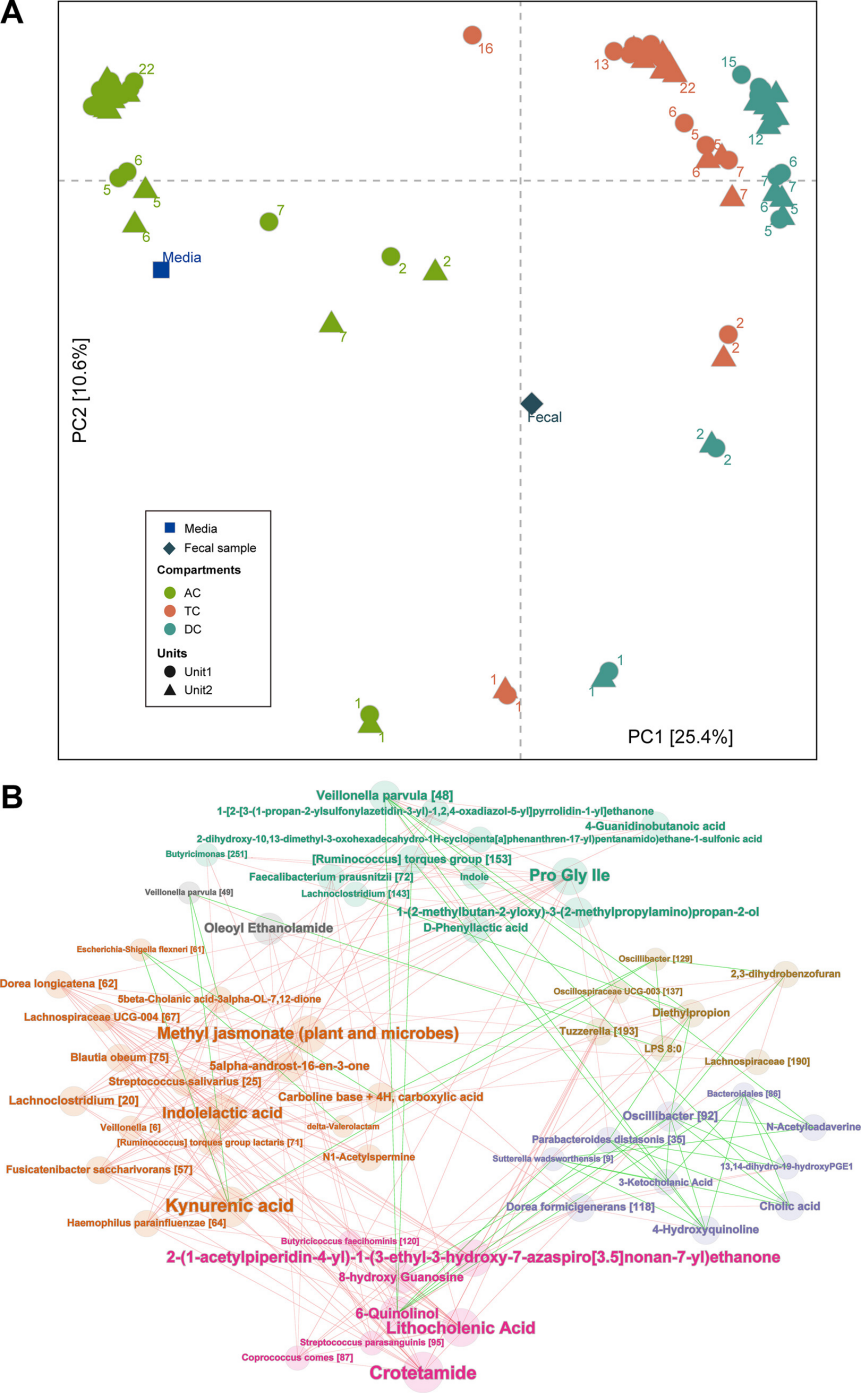
(A) Projection of the first two principal components in the metabolome composition for Exp1. Different compartments follow different trajectories and stabilize around D12, similar to the microbiome compositions in Fig. 2A. (B) Microbe-metabolite bipartite correlation network in the DC compartments for Exp1 identifies six coherent groups. Positive correlations are denoted by green edges and negative correlations by red edges. Coherent groups are identified by different colors for the nodes and node labels. Node size corresponds to its degree. ASVs are indicated by taxonomic annotation followed by ASV ID. Only mass features that were annotated using their MS/MS fragmentation pattern were selected for this network analysis.

In Exp1, we performed untargeted metabolomics analysis on fecal inoculum, fresh media, and supernatants from *in vitro* samples to understand the differences between the exometabolomes from different compartments and to investigate microbe-metabolite interactions. A total of 3,398 *m/z* features in positive mode and 10,448 *m/z* features in the negative mode were detected across all samples (see Materials and Methods for details and Table S1 for the list of mass features). Beta-diversity analysis based on the detected features showed that D1 exometabolome compositions in all three compartments started between fecal and medium metabolite compositions (Fig. 5A). Longitudinal trajectories of the exometabolome compositions were comparable to those of microbiome compositions seen in Fig. 2A—different trajectories for the different compartments stabilizing around D12 at individual stable compositions—reinforcing the connection between the microbiome and exometabolome compositions. Metabolite compositional changes at 1-day intervals further confirmed that the metabolomes stabilized around D12 (Fig. S9). The AC exometabolome was the closest to the medium metabolome, followed by TC and DC exometabolomes, agreeing with the flow between compartments.

10.1128/mSystems.00232-21.9 FIG S9 Metabolite composition differences within each compartment at 1-day intervals suggest stabilization of metabolite composition in Exp1. Metabolite composition differences were estimated by z-scoring log-transformed peak intensities and calculating Euclidean distance between samples. Download FIG S9, PDF file, 0.02 MB. Copyright © 2021 Gnanasekaran et al. 2021 Gnanasekaran et al. https://creativecommons.org/licenses/by/4.0/ This content is distributed under the terms of the Creative Commons Attribution 4.0 International license.

10.1128/mSystems.00232-21.10 TABLE S1 Metabolites identified via MS/MS fragmentation patterns, their features, annotations, and peak intensities in different samples. Download Table S1, XLSX file, 0.2 MB. Copyright © 2021 Gnanasekaran et al. 2021 Gnanasekaran et al. https://creativecommons.org/licenses/by/4.0/ This content is distributed under the terms of the Creative Commons Attribution 4.0 International license.

Microbes in a community interact with each other via resources such as metabolites. Understanding these interactions in an ecological network is crucial for deriving associations between the members of a community (54). The large difference between the DC exometabolome and the medium metabolome suggested that DC harbored the most biochemically active microbial community, at least in terms of secreted metabolites. Hence, we derived a microbe-exometabolite network for DC compartments using 162 mass features annotated based on tandem mass spectrometry (MS/MS) fragmentation and 129 prominent ASVs present at at least three time points. We generated a bipartite correlation network, applied community discovery on this network, and identified 6 different coherent groups (Fig. 5B). These coherent groups are different from the traditional microbe-microbe correlation subnetworks (55), as the stratification here is mediated by exometabolites secreted by microbes. For instance, a traditional correlation network might not have grouped *S. wadsworthensis* [9] with Dorea formicigenerans [118], or *Oscillibacter* [129] with *Lachnospiraceae* [190], as their relative abundance profiles were quite different (Fig. 3A). However, they were connected in our bipartite network via exometabolites that correlate with both (Fig. 5B).

Among the exometabolites that were strongly correlated with ASVs, we identified several that were relevant for host health. For instance, kynurenic acid, a neuroprotective (56) and anti-inflammatory (57) metabolite, was positively correlated with Escherichia-Shigella flexneri [61] and *V. parvula* [49] and negatively correlated with Dorea longicatena [62], Fusicatenibacter saccharivorans [57], and *Lachnospiraceae* UCG-004 [67] (Fig. 5B). Kynurenine, the substrate for kynurenic acid, is produced by selective cleavage of tryptophan by the enzyme tryptophan 2,3-dioxygenase, found in mammals and bacteria (58, 59). The positive correlation of kynurenic acid to Escherichia*-*Shigella flexneri [61] is in agreement with previous findings that it can be produced by Escherichia coli (60). Another tryptophan-derived metabolite, indole, known for its role in reducing intestinal inflammation, exhibited no positive correlations to any ASVs but only negative correlations to Bifidobacterium dentium [152], *Lachnoclostridium* [133], and *Butyricicoccus* [163] (Fig. 5B). This was very surprising, as indole is a prevalent gut microbial metabolite known to be produced by many gut bacteria that harbor the tryptophanase gene (61). However, we observed positive correlations of multiple ASVs to indolelactate in our network, indicating the possibility that most of the free indole might have been converted to other metabolites, such as indolelactate. Various studies have shown that indolelactate is produced mainly by *Bifidobacterium* spp. (62–64), and the negative association of *B. dentium* [152] with indole could indicate the conversion of free indole to indolelactate.

Among bile acids and their derivatives, we detected cholic acid, lithocholenic acid, taurocholic acid, and 5β-cholanic acid-3α-ol-7,12-dione. Cholic acid, an unconjugated primary bile acid synthesized from cholesterol in the human liver, was part of the pancreatic juice fed to the colon compartments in abundance. Hence, its positive correlation with multiple highly abundant bloomer and survivor ASVs such as *S. wadsworthensis* [9] and Parabacteroides distasonis [35] could be due to the continuous accumulation of unmetabolized cholic acid in the media (Fig. 5). We found 27 ASVs negatively correlated with lithocholenic acid, potentially suggesting the conversion of lithocholenic acid to secondary bile acids such as lithocholic acid, isolithocholic acid, ketolithocholic acid, ursodeoxycholic acid, and chenodeoxycholic acid (65). However, we could not confirm the production of these secondary bile acids, as we did not identify these bile acids unambiguously in our metabolomics analysis.

### Conclusions.

By evaluating the longitudinal adaptation and temporal variation of two fecal microbial communities in a multistage fecal fermentor, our study sheds light on the dynamics of a complex gut bacterial community grown *in vitro* under different colonic conditions. Specifically, our study also illuminates the dynamics of some of the interesting and clinically relevant species in a complex *in vitro* community. For instance, we could study the dynamics of (i) *A. muciniphila*, a well-known probiotic microbe that plays a significant role in regulating various host functions (66, 67); (ii) *S. wadsworthensis*, which is associated with inflammation (68); (iii) *B. wadsworthia*, which is associated with inflammation and intestinal barrier dysfunction (69); (iv) P. distasonis, which is associated with the alleviation of obesity and colitis (70); and (v) *F. prausnitzii*, which has been negatively correlated with various disease conditions such as inflammatory bowel disease, irritable bowel syndrome, colorectal cancer, obesity, and celiac disease (47, 71). These results imply that *in vitro* fermentors are suitable for growing and characterizing clinically relevant microbes, both beneficial and detrimental to human health, as part of a complex community similar to the human gut microbiota. Distinct microbial compositions in the two experiments suggest that *in vitro* fermentors can maintain the individuality of fecal microbiota rather than converging on a fermentor-specific composition. Nevertheless, using an *in vitro* gut fermentor is not devoid of limitations: in both experiments, less than one-third of fecal ASVs managed to persist in the fermentor after stabilization. This implies that the combination of conditions and feed used here may not be favorable for all members of the gut microbiota. At the same time, another 33 ASVs in Exp1 and 45 ASVs in Exp2 that were not detected in the fecal microbiome (at a detection threshold of 6.2 million cells per g feces) persisted in the fermentor after stabilization, suggesting that *in vitro* fermentors can enrich the hidden biodiversity in fecal microbiota.

The successful growth of any desired microbe in an *in vitro* fermentor that harbors a complex and diverse community composition strongly relies on the symphony of both ecological and molecular forces. The diversity of species also means intense competition, but also potentially mutually beneficial relationships. Hence, it is crucial to have the presence of a favorable ecological niche that provides beneficial interactions among the candidate members of a community and the microbes of interest. This can be addressed by modulating the feed and by introducing engineered synthetic communities comprising commensals and mutualists wherein, most importantly, competitors are eliminated.

## MATERIALS AND METHODS

### SHIME setup design, inoculation, and sample collection.

All experiments were carried out using the *in vitro* digestion model SHIME, specifically the luminal SHIME (L-SHIME). The setups in our experiment were adapted from the TwinSHIME and QuadSHIME models (Fig. S1) (14). Each unit in Exp1 comprised a succession of five compartments (reactors) that simulated stomach (ST), small intestine (SI), ascending colon (AC), transverse colon (TC), and descending colon (DC), while the Exp2 was performed with a combined stomach and small intestine (ST+SI) compartment, an AC compartment and a DC compartment (21). All compartments were continuously stirred at 300 rpm, and the temperature was kept constant at 37°C. Anaerobic conditions were maintained by flushing all compartments with 100% N_2_. The first two compartments of Exp1 and the first compartment of Exp2 were based on the fill-and-draw principle simulating stomach and small intestine, with a peristaltic pump adding 140 ml SHIME nutritional medium (ProDigest BVBA, Ghent, Belgium) and 60 ml pancreatic and bile liquid (ProDigest BVBA, Ghent, Belgium) in three cycles a day with an 8-h interval between cycles. The SHIME nutritional medium comprised arabinogalactan (1.2 g/liter), pectin (2 g/liter), xylan (0.5 g/liter), glucose (0.4 g/liter), yeast extract (3 g/liter), special peptone (1 g/liter), mucin (3 g/liter), l-cysteine-HCl (0.5 g/liter), and starch (4 g/liter). The pancreatic and bile liquid contained NaHCO_3_ (12.5 g/liter), oxgall (6g/liter), and pancreatin (0.9 g/liter). The last three compartments of Exp1 and the last two compartments of Exp2 were continuously stirred compartments simulating colonic conditions. In Exp1 and Exp2, for the colon compartments, the volume (V) and the pH were maintained as follows: for Exp1, the AC pH was 5.6 to 5.9 and V was 500 ml, the TC pH was 6.15 to 6.4 and V was 800 ml, and the DC pH was 6.6 to 6.9 and V was 600 ml; for Exp2, the AC pH was 5.6 to 5.9 and V was 500 ml and the DC pH was 6.6 to 6.9 and V was 800 ml.

For the inoculation of colon compartments, the fecal inoculum was prepared from feces obtained from two anonymous human donors with no history of antibiotic use 6 months prior to the study. For this purpose, we used fresh fecal samples (less than 1 h after voiding). Fecal materials were mixed with anaerobic phosphate buffer in a 20% (wt/vol) proportion. The anaerobic phosphate buffer contained K_2_HPO_4_ (8.8 g/liter), KH_2_PO_4_ (6.8 g/liter), sodium thioglycolate (0.1 g/liter), with the pH adjusted to 7, and was boiled to make it anaerobic. Prior to use, sodium dithionite (15 mg/liter) was added to the buffer and stored at room temperature. The fecal suspension was then homogenized using the Stomacher 400 circulator (Seward, UK) at 300 rpm for 10 min. Upon homogenization, the suspension was centrifuged for 2 min at 500 × *g* to separate the inoculum (supernatant) from the debris. The inoculation was performed with the resulting fecal inoculum in 5% (vol/vol) proportions in all colon compartments.

For Exp1, samples (1 ml) were collected from AC, TC, and DC compartments in both units on days 1, 2, 5, 6, 7, 12, 13, 15, 16, 17, 20, 21, and 22, and for Exp2, samples were collected every second day for AC and DC compartments from day 1 until day 31. All collected samples were centrifuged at 6,000 rpm for 5 min to separate bacterial cells from the culture. In Exp1, for days 2, 7, 12, 17, and 22 as well as for fecal samples, triplicate sampling was performed. Upon centrifugation, both the bacterial pellet and culture supernatants were stored at −80°C for further analysis.

### DNA extraction, 16S rRNA gene amplicon sequencing.

Genomic DNA from the bacterial pellets was extracted using a NucleoSpin Soil kit (Macherey-Nagel, Duren, Germany) following the manufacturers’ instructions with a modification in the sample lysis step. For efficient lysis, the bacterial pellets were resuspended in the optional enhancer SX solution and SL1 buffer and homogenized using TissueLyser II (Qiagen, Hilden, Germany) at a speed of 30 oscillations/s for 5 min. The concentrations and quality of DNA were evaluated using a NanoDrop system and Qubit Fluorometer/microplate reader (Thermo Fisher Scientific, USA). 16S rRNA gene amplicon sequencing targeting the V4 variable region was performed using the Illumina HiSeq 2500 system, producing 2 × 250-bp paired-end reads. Altogether, 3,031,524 high-quality paired-end reads (median, 20,760 per sample) from 3 fecal inoculum replicates and 143 *in vitro* samples in Exp1 and 8,117,537 high-quality paired-end reads (median, 231,733 per sample) from one fecal inoculum sample and 32 *in vitro* samples in Exp2 were generated.

### Total cell counting.

Cell counting was carried out using the Quantom Tx microbial cell counter (Logos Biosystem, South Korea). The bacterial pellets were resuspended in sterile SHIME nutritional media, and the samples were diluted 10 times. Ten microliters of diluted samples was mixed well with 1 μl of Quantom total cell staining dye, 1 μl of Quantom total cell staining enhancer, and 8 μl of Quantom cell loading buffer I. From the resulting mixture, 6 μl was loaded on a Quantom M50 cell counting slide and centrifuged at 300 × *g* for 10 min in a Quantom centrifuge. Then, the samples were counted with the Quantom Tx microbial cell counter with the following parameters: light intensity, level 5, size gating, ∼0.3 to 50 μm; roundness, 25%; declustering level, 10; and detection sensitivity, 9.

### Bioinformatics and statistical analysis.

All analyses were performed in R software (v3.6.2).

### (i) Sequence quality control.

We used the DADA2 v1.16 R package (32) to process the 2 × 250-bp Illumina HiSeq amplicon sequencing reads representing the V4 region of 16S rRNA genes. Primers were removed from raw reads; and reads were filtered and trimmed using the parameter truncLen=c(200,160).

After this, 20,000 high-quality filtered and trimmed reads were randomly selected to remove the confounding effect of sequencing depth between the two experiments. The error-rate-learning step was performed individually for each experiment. Then, reads were dereplicated and merged. Chimeras were identified and removed from the amplicons, leaving 424 unique amplicon sequence variants (ASVs).

### (ii) Taxonomic classification of ASV sequences.

Taxonomic classification of ASV sequences was performed using the Silva 138 database (72) following recommended procedure from DADA2 developers: using the assignTaxonomy function (with silva_nr_v138_train_set.fa), which assigns taxonomy up to the genus level, followed by the addSpecies function (with silva_species_assignment_v138.fa), which uses exact sequence matching to assign species.

### (iii) Full-length 16S rRNA gene sequencing and taxonomic assignment for DC samples.

To improve species-level assignment, we sequenced near-full-length 16S rRNA gene regions from 29 samples from Exp1 (14 DC samples each from unit 1 and unit 2; 1 fecal sample) using PacBio sequel II circular consensus sequencing (CCS) technology using the manufacturer’s recommendation (number 101-599-700, version 03 [February 2020]; Pacific Biosciences, USA). We derived 832,879 high-quality circular consensus sequences (median, 26,555 sequences per sample) using the CCS program v4.2.0 with the following parameters: –min-snr = 3.75 –max-length = 7000 –min-length = 1200 –min-passes = 20 –min-rq = 0.9995 (73). We then generated unique full-length 16S rRNA gene sequences using two approaches: (i) ASVs using the workflow recommended by DADA2 developers (74) and (ii) zero-radius operational taxonomic units (ZOTUs) using the UNOISE algorithm (75). To ensure high reliability, we derived sequences found by both approaches as the full-length ASVs. We then taxonomically annotated these ASVs using the assignSpecies function (with silva_nr99_v138_wSpecies_train_set.fa).

### (iv) Improving taxonomy assignment of V4 region ASVs using full-length ASVs.

To improve the taxonomic resolution of the V4 ASVs, we aligned them to the full-length ASVs and transferred better-resolved taxonomy from the latter if a V4 ASV aligned either to only one full-length ASV or to multiple full-length ASVs with identical taxonomy. This increased species-level resolution from 95 to 121. Finally, ASVs without phylum assignment were discarded.

### (v) Alpha- and beta-diversity analyses.

Alpha- and beta-diversity analyses were performed using the phyloseq package (v1.30.0). Alpha diversity between colon compartments was compared by applying the Wilcoxon signed-rank test using ASV richness and the Shannon index. Beta diversity analysis was done by calculating the Jensen-Shannon distance.

### (vi) Heat map visualization.

For visualization purposes, only prominent ASVs present in at least three time points (including fecal samples) were considered. Heat map rows were ordered based on hierarchical clustering. For visualization, ASV relative abundance was converted to read counts by multiplying with approximate average sequencing depth (20,000 reads) and normalized in log scale by the formula log_10_ (1+ *x_i_*)/max {log_10_ (1+ *x_i_*)}, where *x_i_* is the read count for a given ASV in sample *i*.

### (vii) Network analysis.

Correlations (between ASV relative abundances and metabolite abundances) were calculated using Spearman rank correlation. In the bipartite microbe-metabolite network, each node corresponds to either an ASV or a mass feature detected in the DC compartment. Edges in this network connect only ASVs with metabolites (i.e., no links within metabolites themselves or within ASVs). An edge between two nodes corresponds to a correlation, positive or negative, between the corresponding mass feature and ASV, calculated using Spearman’s correlation. In the visualized network, we considered absolute correlation values greater than 0.6 with statistical significance (*P* ≤ 0.001). In the visualizations, node size corresponds to the degree of the node in the graph, and node color corresponds to its community membership when applying community discovery on the graph using the Louvain method (76).

### (viii) Longitudinal trends on cell count.

Since raw cell counts were noisy, cell count longitudinal trends were calculated using a third-degree polynomial model as implemented in the R function lm. The R function predict was applied to extract the values predicted by the lm function, which were used for further analysis.

### Metabolomics analysis.

**(i) Chemicals and reagents.** High-performance liquid chromatography (HPLC)-grade water, acetonitrile, and methanol (MeOH) were purchased from Honeywell (Charlotte, NC, USA). Labeled standards were acquired from Cambridge Isotope Laboratories, Inc. (Tewksbury, MA, USA). Electrospray ionization low-concentration (ESI-L) tuning mix was purchased from Agilent Technologies (Santa Clara, CA, USA). Formic acid (≥99.5%), Optima liquid chromatography-mass spectrometry (LC-MS) grade, was purchased from Fisher Chemical (Pittsburgh, PA, USA).

**(ii) Sample preparation for metabolite extraction.** From the supernatants obtained from SHIME compartments, 50 μl of supernatants was treated with 100 μl of 50% MeOH. For quality control and normalization, 50 μl of two internal labeled standard mix of chenodeoxycholic acid-d4 (1 mg/liter) and l-phenylalanine-^13^C_9_,^15^N (1 mg/liter) in MeOH was added to the extraction solvent. Samples were vortexed and precipitated on ice for 30 min. After precipitation, extracts were centrifuged at 10,000 rpm at 4°C for 3 min for protein precipitation and metabolite extraction. The supernatants containing the polar metabolites were collected in LC vials for LC-MS analysis (77).

**(iii) Quality control.** A pooled sample (quality control sample [QC]) of the reconstituted extracts was prepared by mixing equal volumes of each sample. The pooled sample was further diluted with MeOH at 1:1 and 1:2. To condition the column, the QC sample dilution series was injected at least 3 times before initiating the run. Then, the sample was reinjected every 10 sample injections and at the end of the run to assess instrument stability and analyte reproducibility. An equal volume of a blank sample consisting of 100% MeOH was randomly inserted into the real sample queue to be processed as a needle wash and to equilibrate the column, as well as to avoid contamination among real samples. The analytical reproducibility in terms of detected intensities of detected *m/z* features was evaluated by calculating the coefficient of variation (CV) of detected peaks in QC samples and by visualizing the tight clustering of QC samples in principal-component analysis (PCA) (78).

**(iv) Analysis of metabolites by UHPLC–TOF-MS.** Metabolomics profiling was performed using an ultrahigh-performance liquid chromatography (UHPLC) system (Agilent 1290 Infinity II) connected to a Bruker timsTOF Pro instrument equipped with a trapped-ion mobility spectrometer (TIMS) coupled to a hybrid quadrupole time-of-flight mass spectrometer (TOF-MS) (Bruker, Bremen, Germany). Ions were generated in the positive and negative electrospray ionization modes. The samples were randomized and analyzed using reversed-phase Acquity UPLC HSS T3 columns, 100 Å, 1.8 μm, 2.1 mm by 50 mm (Waters, Milford, MA). The column and autosampler temperatures were maintained at 40°C and 10°C, respectively. Solvent A, consisting of 0.1% formic acid in water, and solvent B, consisting of 0.1% formic acid in acetonitrile and propanol (3:1, vol/vol), were used as mobile phases. The injection volume and flow rate were 5 μl and 0.4 ml/min, respectively. The UPLC gradient was programmed as follows: 0 to 10% B over 0 to 2 min, 99% B (2 to 9 min), 0.1% B (9 to 10 min). The ESI source used 10 liters/min of drying gas at a temperature of 220°C. The ESI was set at a 3,500-V capillary voltage and a 300-kPa nebulizer pressure. Detection of the mass/charge ratio (*m/z*) of ions was set from 50 to 1,000 over 10 min. To facilitate the compound identifications, QC samples were analyzed by auto-MS/MS in positive and negative electrospray ionization modes. The absolute threshold was set to 1,000 counts. MS and MS/MS spectrum acquisition rates were set to 4 Hz, with a total cycle time of 1 s for precursor ion collection. The collision energy varied between 10 eV and 60 eV.

**(v) MS data processing.** Data acquisition was performed with otofControl version 6.0 and Bruker Compass HyStar version 5.0 (Bruker Daltonics, Bremen, Germany), and data processing was performed with Bruker Compass Data Analysis 5.2 software and MetaboScape version 5.0 (Bruker Daltonics, Bremen, Germany). Molecular feature selection, bucketing, filtering, and scaling were performed by MetaboScape to generate the peak lists from MS and MS/MS spectra. An internal calibrant of Na format injected at the beginning of each analysis was used to calibrate the acquired MS and MS/MS data in MetaboScape.

For identification of metabolites, the *m/z* features obtained from MS analysis were matched to the Human Metabolome Database (HMDB) (https://hmdb.ca). The positive and negative ion adducts [M + H]^+^, [M + NH_4_]^+^, [M + Na]^+^, [M+H − H_2_O]^+^, [M+K]^+^, [M + Na − 2H]^−^, [M + Cl]^−^, and [M − H]^−^ were used during annotation, with a confidence limit of 10 ppm to increase sensitivity in the matching of compounds (66). The MS/MS spectra in MetaboScape were annotated using SmartFormula and by comparing the spectra with previously created MS/MS spectral libraries, such as Bruker HMDB Metabolite Library, Bruker MetaboBASE Personal Library 2.0, Bruker MetaboBASE Personal Library 3.0, MoNA, and MSDIAL-TandemMassSpectralAtlas, with a confidence limit of 5 mDa for parent mass tolerance (79). The complete list of the mass features and their intensities for different time points is available in the supplemental material (Table S1).

**(vi) Principal-component analysis.** Metabolite peak intensity data normalization was performed as follows: (i) log transformation using ln⁡(1+x) to avoid taking log of 0, (ii) z-scoring (mean centered and divided by the standard deviation of each variable). PCA was performed on normalized data utilizing Euclidean distance.

### Data availability.

Sequencing reads have been deposited at NCBI Short Read Archive under BioProject identifier PRJNA687518. Metabolomics data have been deposited in the MetaboLights database under study identifier MTBLS2531. Processed microbiome summary data are available as an R phyloseq object at http://arumugamlab.org/SuppData/Gnanasekaran_et_al_2021_microbiome_adaptation/.
