# Supplementary figures and images for: Ecological Adaptation and Succession of Human Fecal Microbial Communities in an Automated In Vitro Fermentation System
Source: mSystems. 2021 Jul 27;6(4):e00232-21. doi: 10.1128/mSystems.00232-21 (PMC8409738; doi:10.1128/mSystems.00232-21)

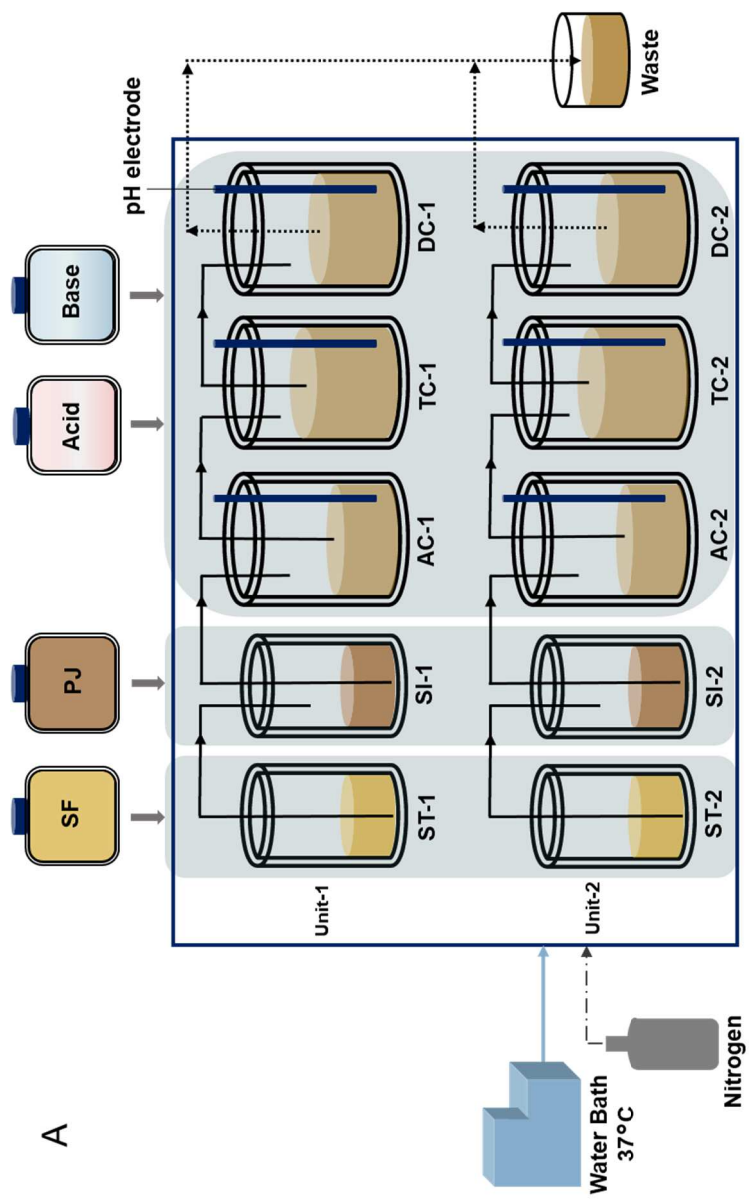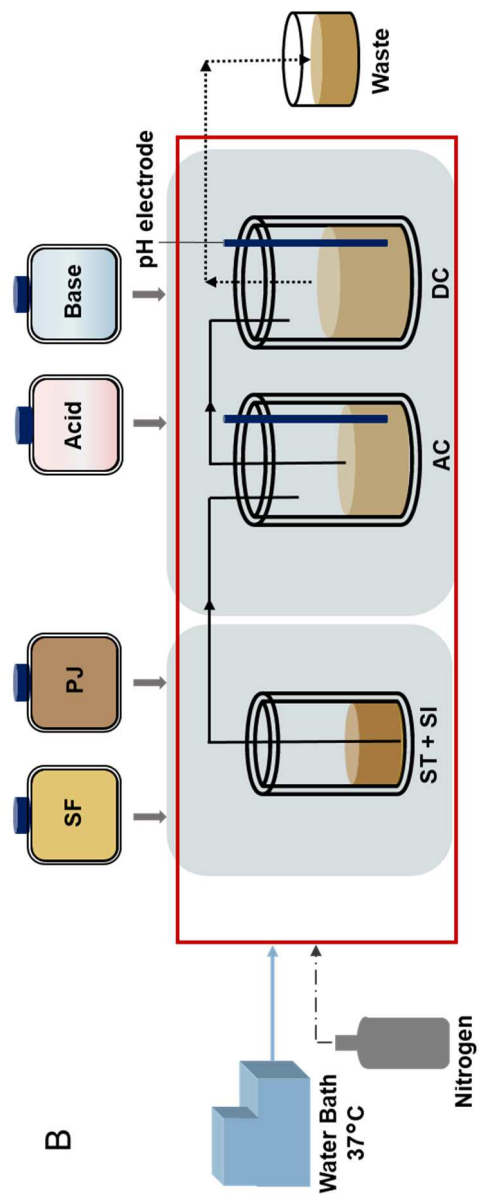

Supplement: FIG S1 [file msystems.00232-21-sf001.pdf]

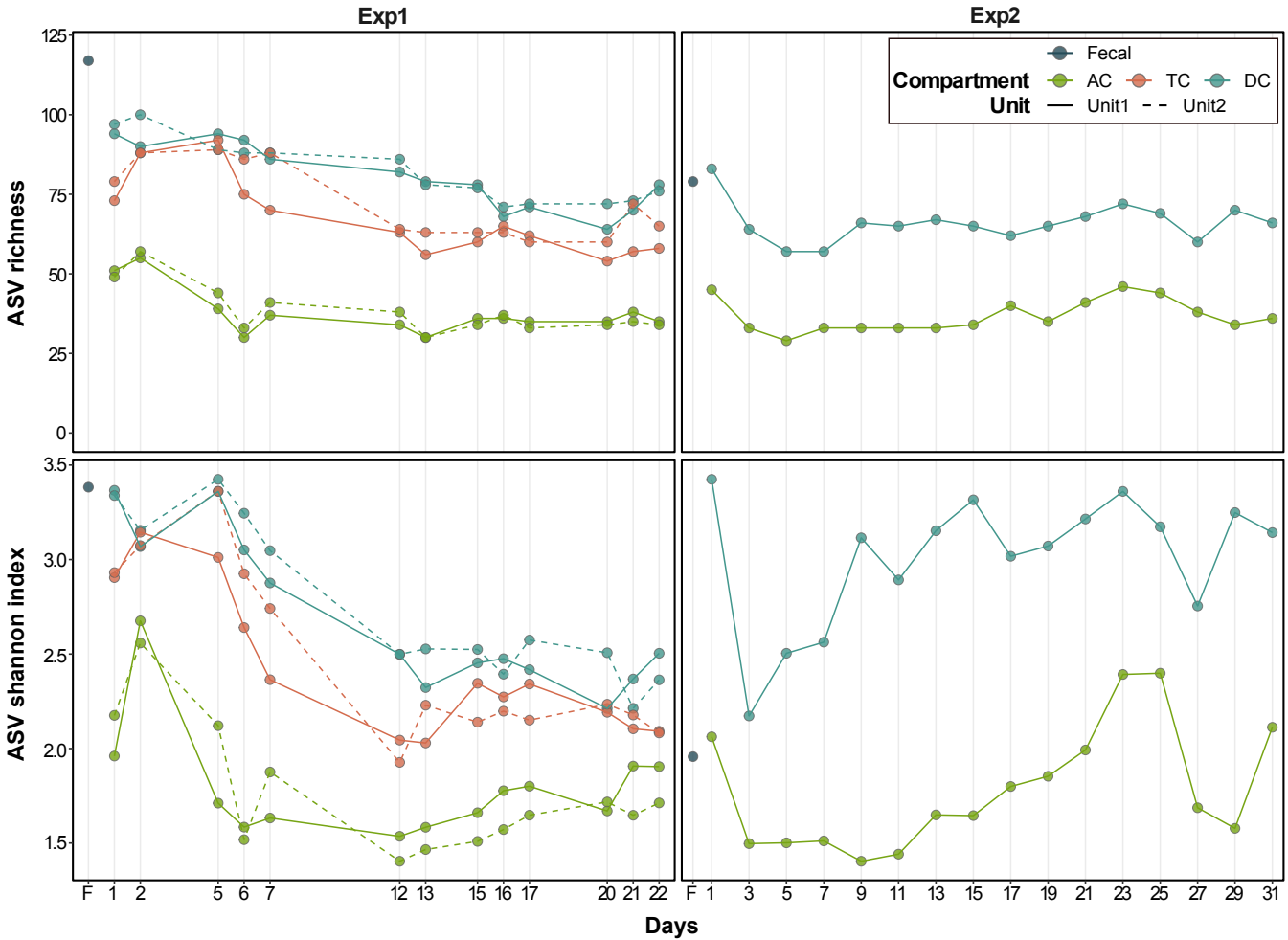

Supplement: FIG S2 [file msystems.00232-21-sf002.pdf]

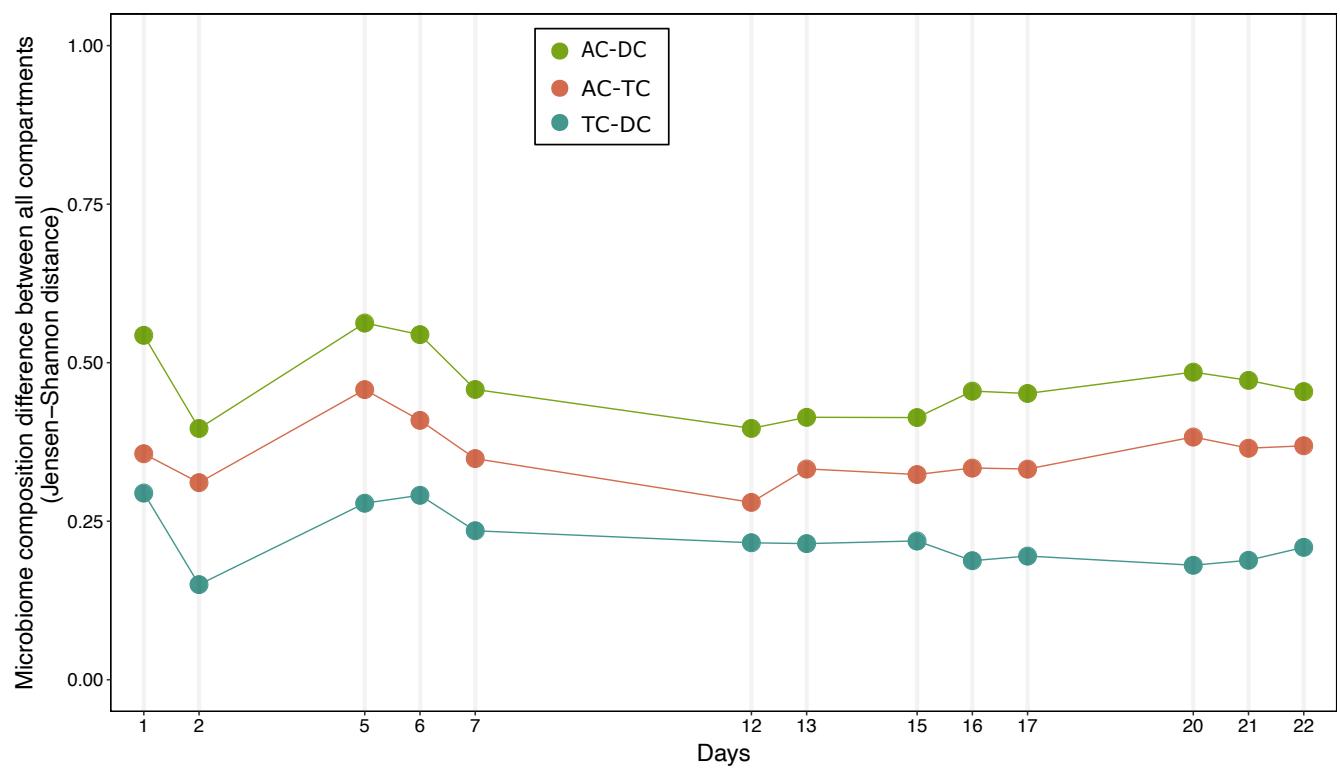

Supplement: FIG S4 [file msystems.00232-21-sf004.pdf]

A

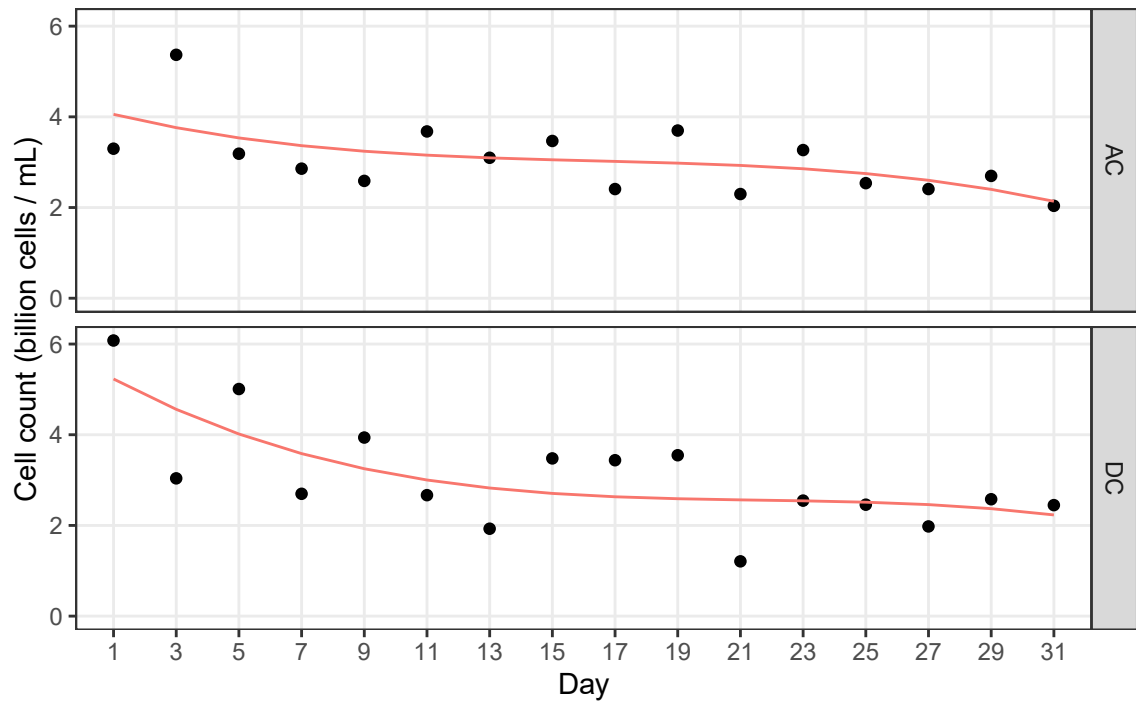

B

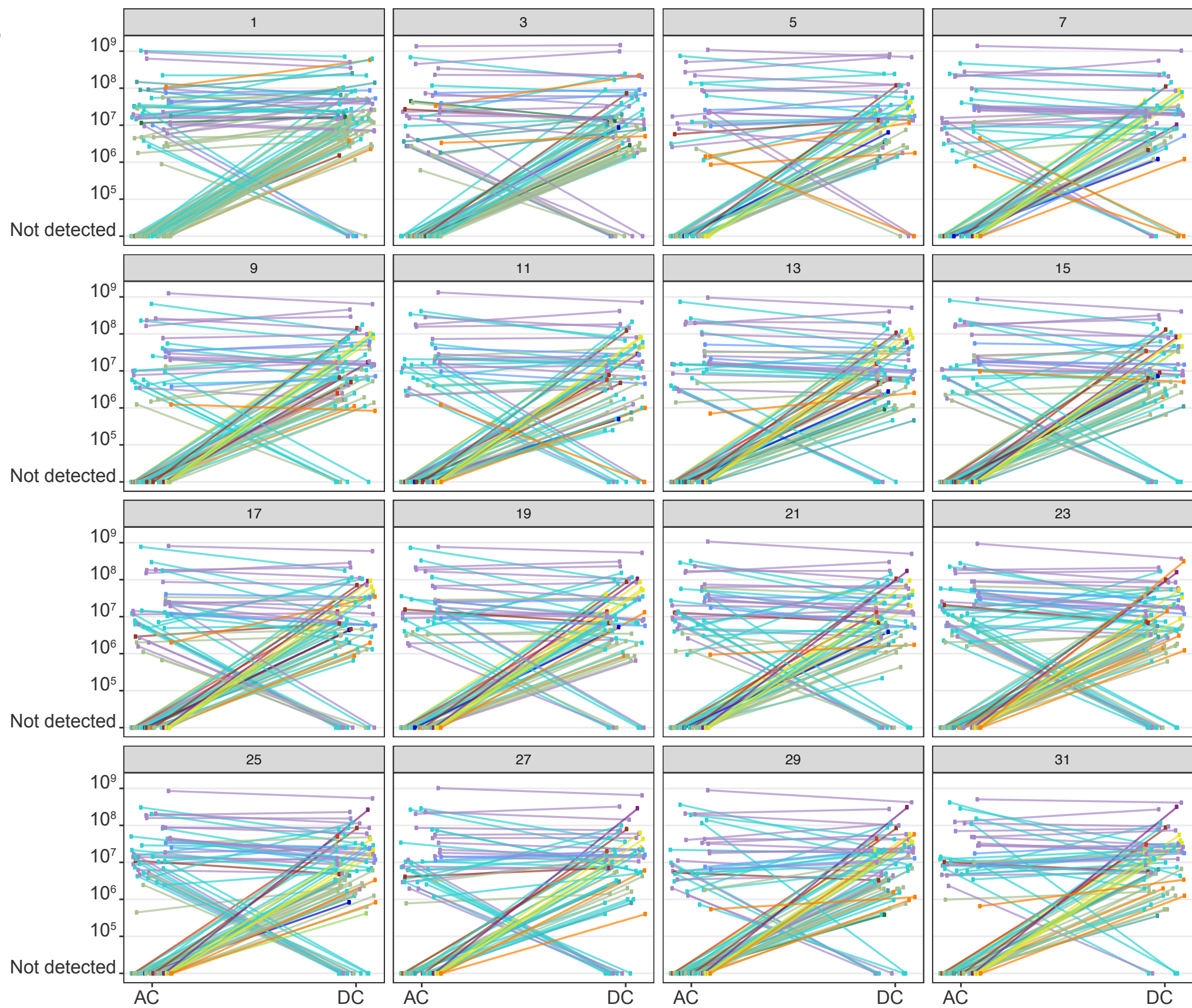

C

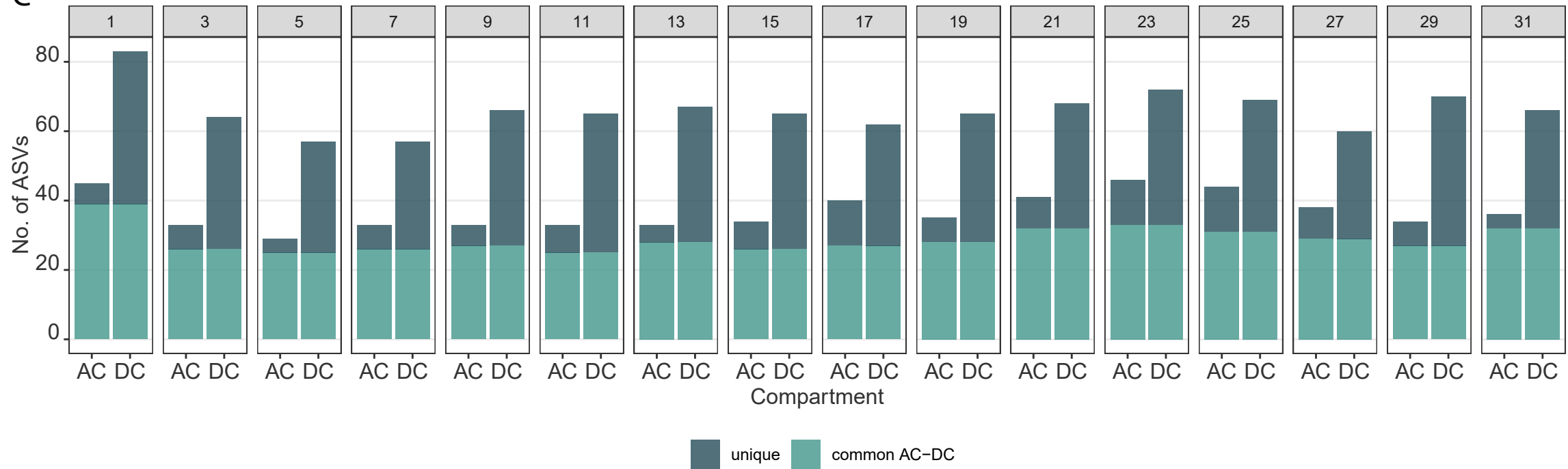

D

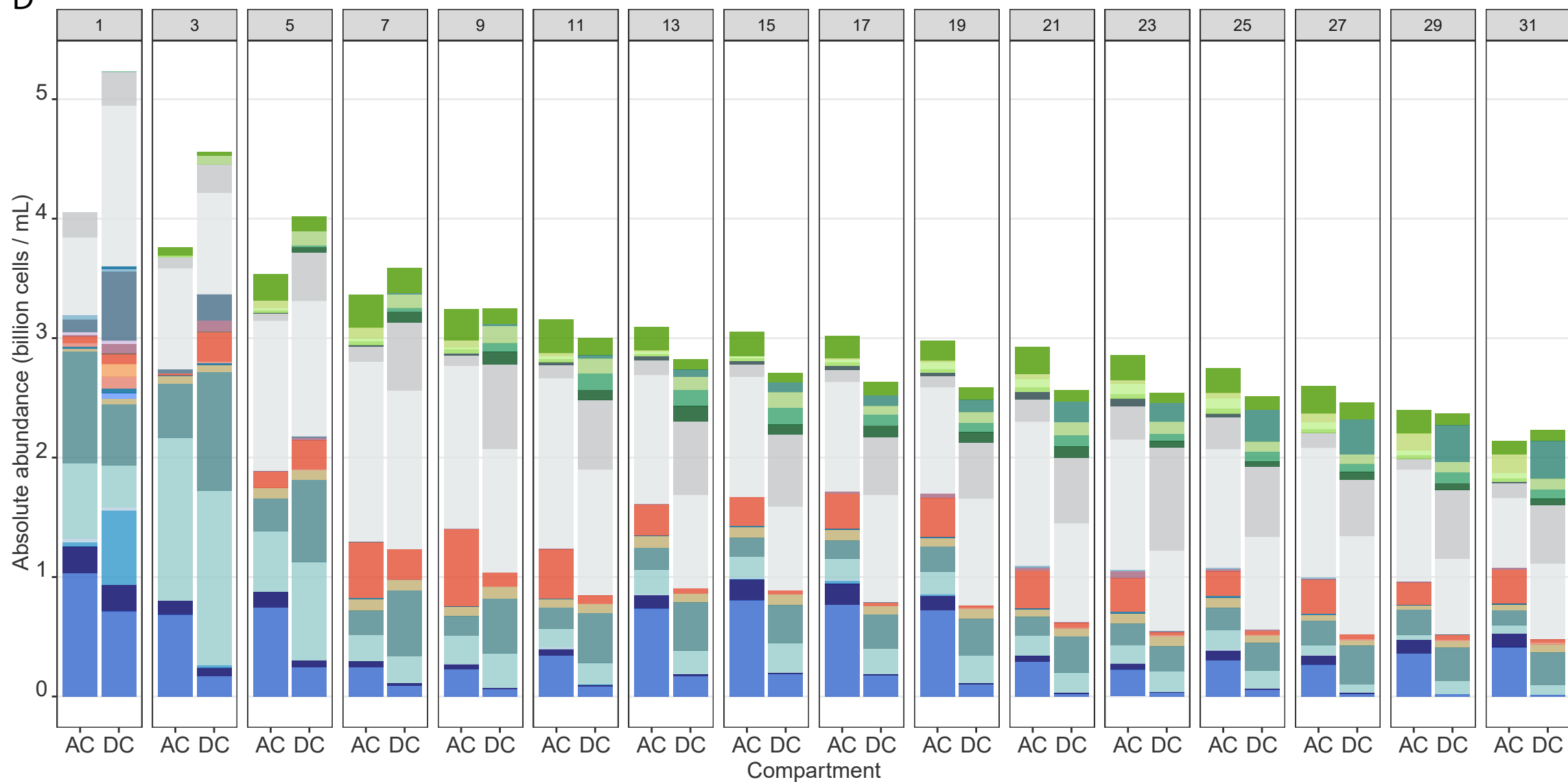

Supplement: FIG S5 [file msystems.00232-21-sf005.pdf]

A

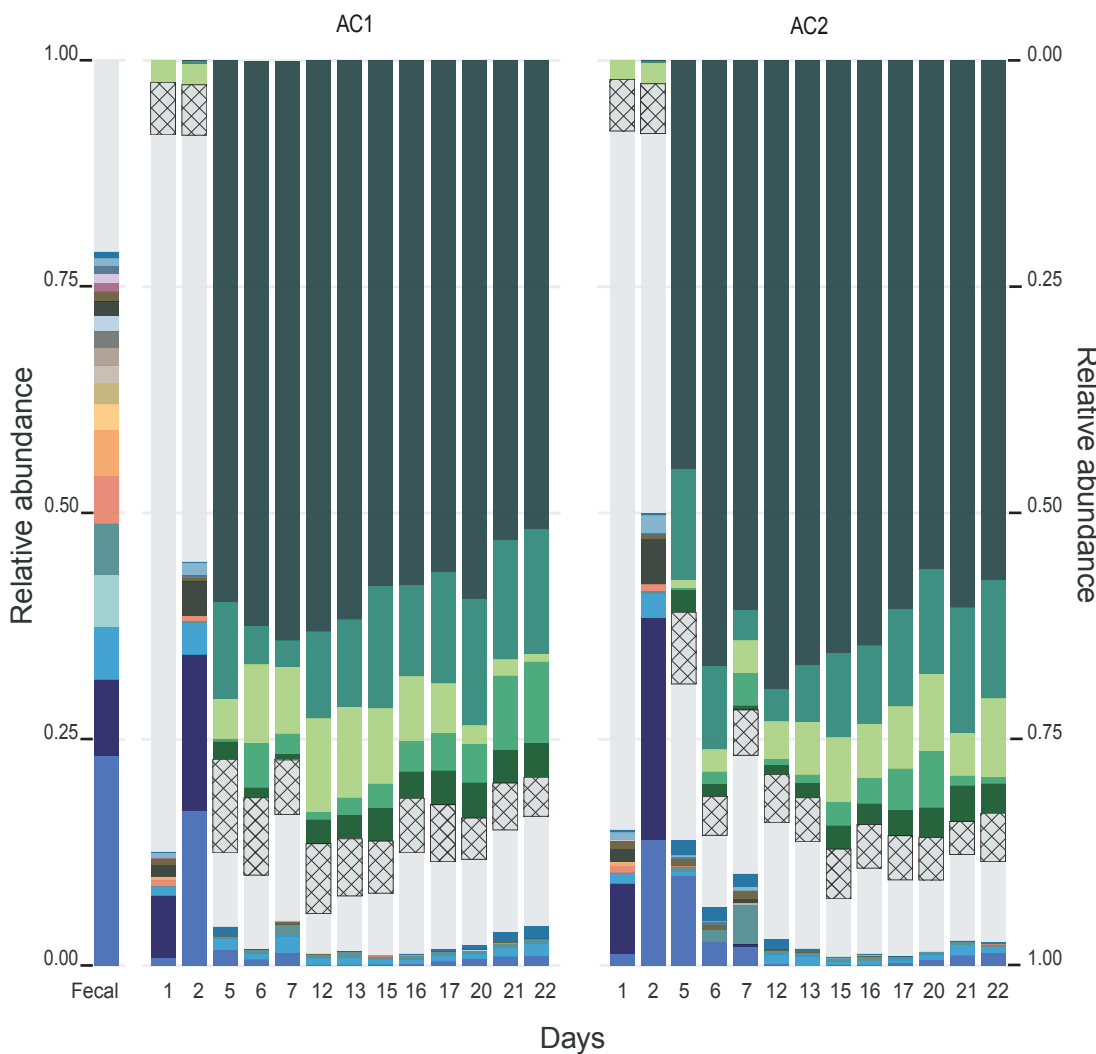

B

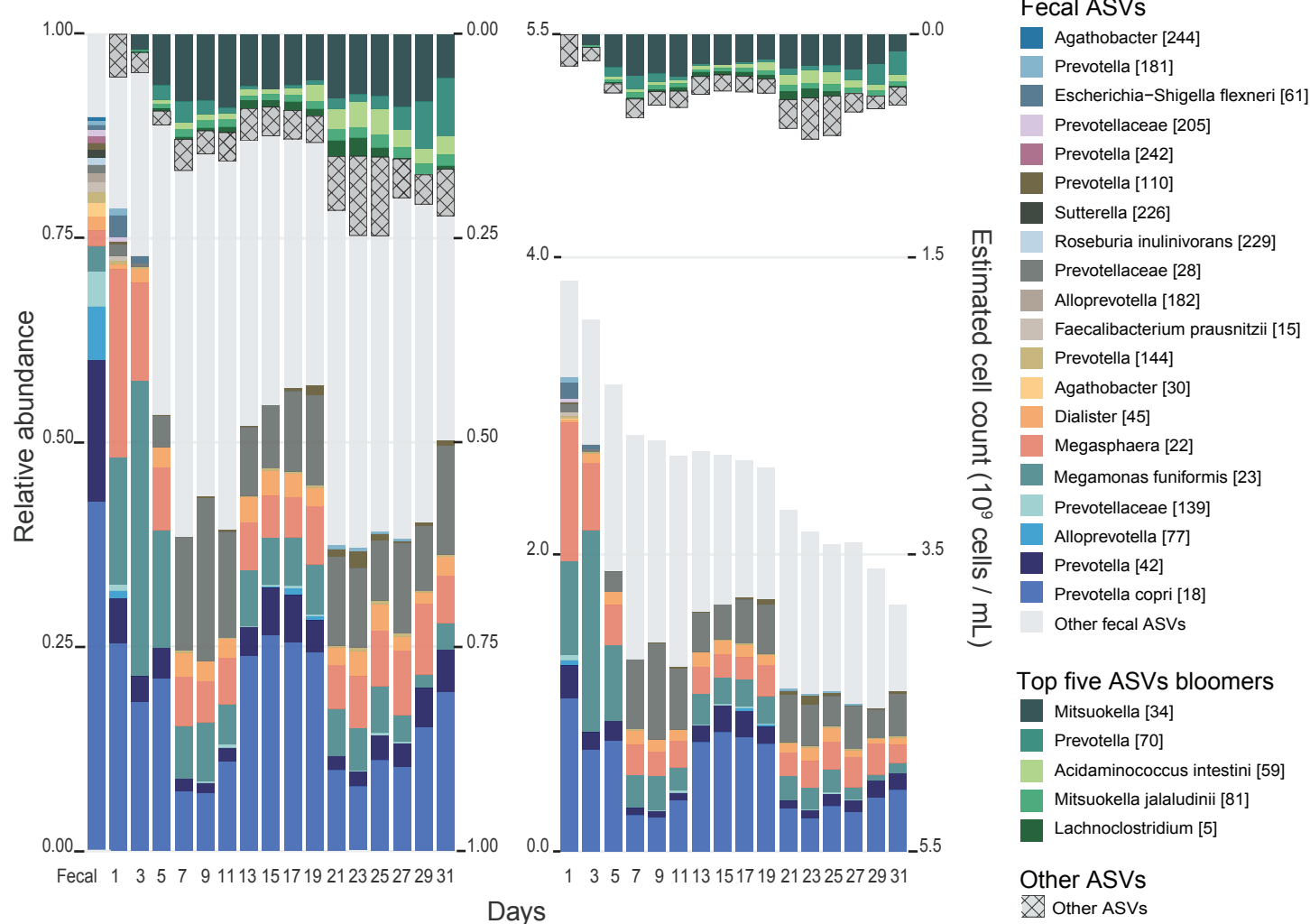

C

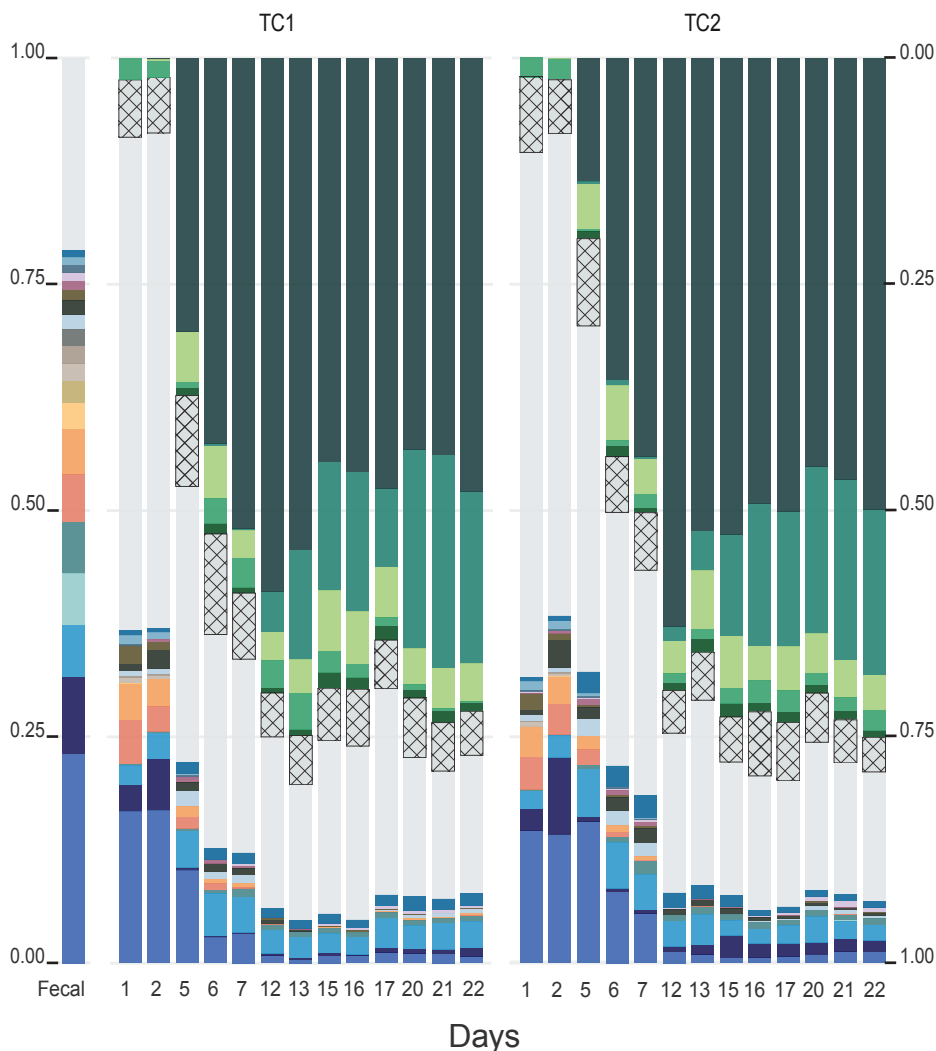

Supplement: FIG S7 [file msystems.00232-21-sf007.pdf]

Study    ···· Exp1    — Exp2    Compartment\_Unit    ● Fecal    ● DC1    ● DC2

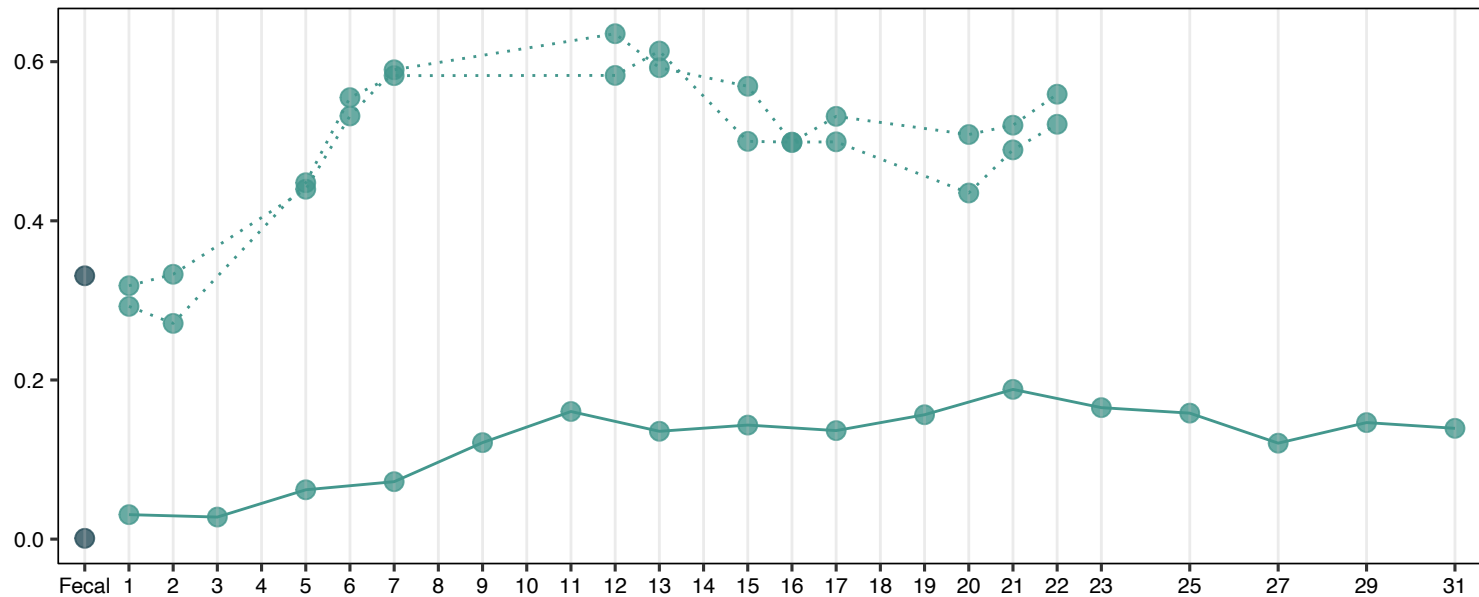

Supplement: FIG S8 [file msystems.00232-21-sf008.pdf]

Metabolite composition difference

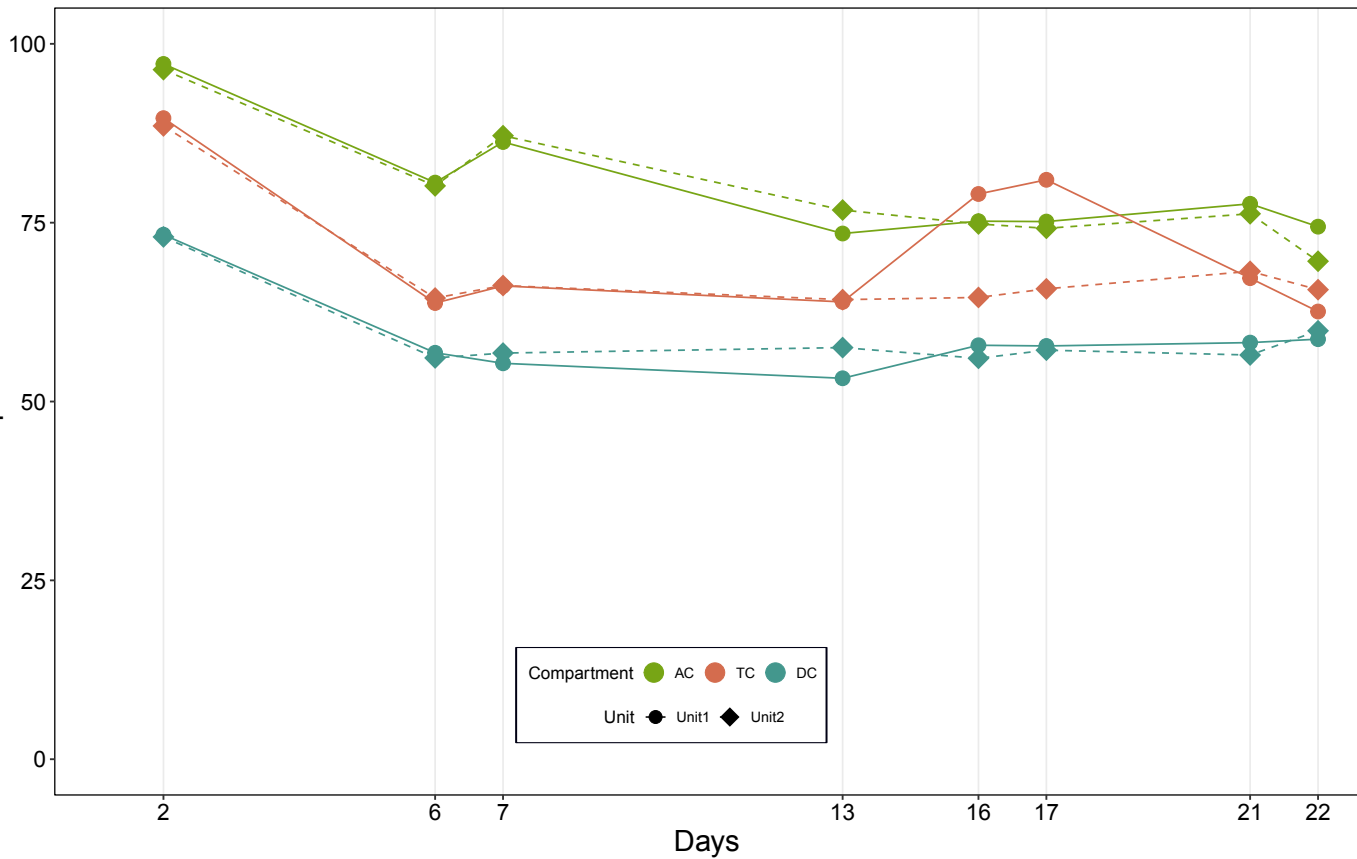

Supplement: FIG S9 [file msystems.00232-21-sf009.pdf]
