# Supplementary material for: Ecological Adaptation and Succession of Human Fecal Microbial Communities in an Automated In Vitro Fermentation System
Source: mSystems. 2021 Jul 27;6(4):e00232-21. doi: 10.1128/mSystems.00232-21 (PMC8409738; doi:10.1128/mSystems.00232-21)
Supplement: FIG S3 [file msystems.00232-21-sf003.pdf]

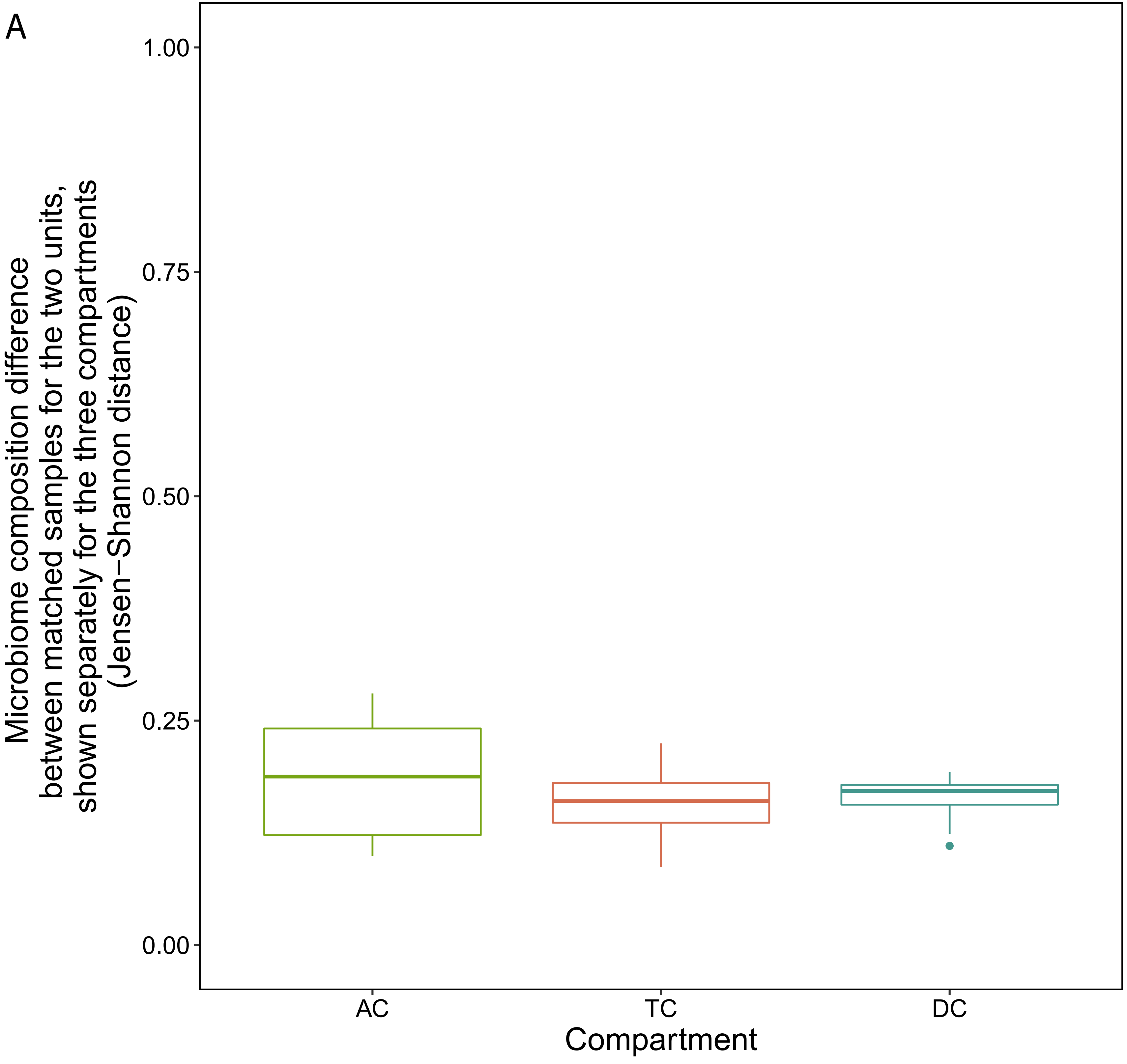

A
1.00
0.75
0.50
0.25
0.00
Microbiome composition difference
between matched samples for the two units,
shown separately for the three compartments
(Jensen−Shannon distance)
AC
TC
DC
Compartment

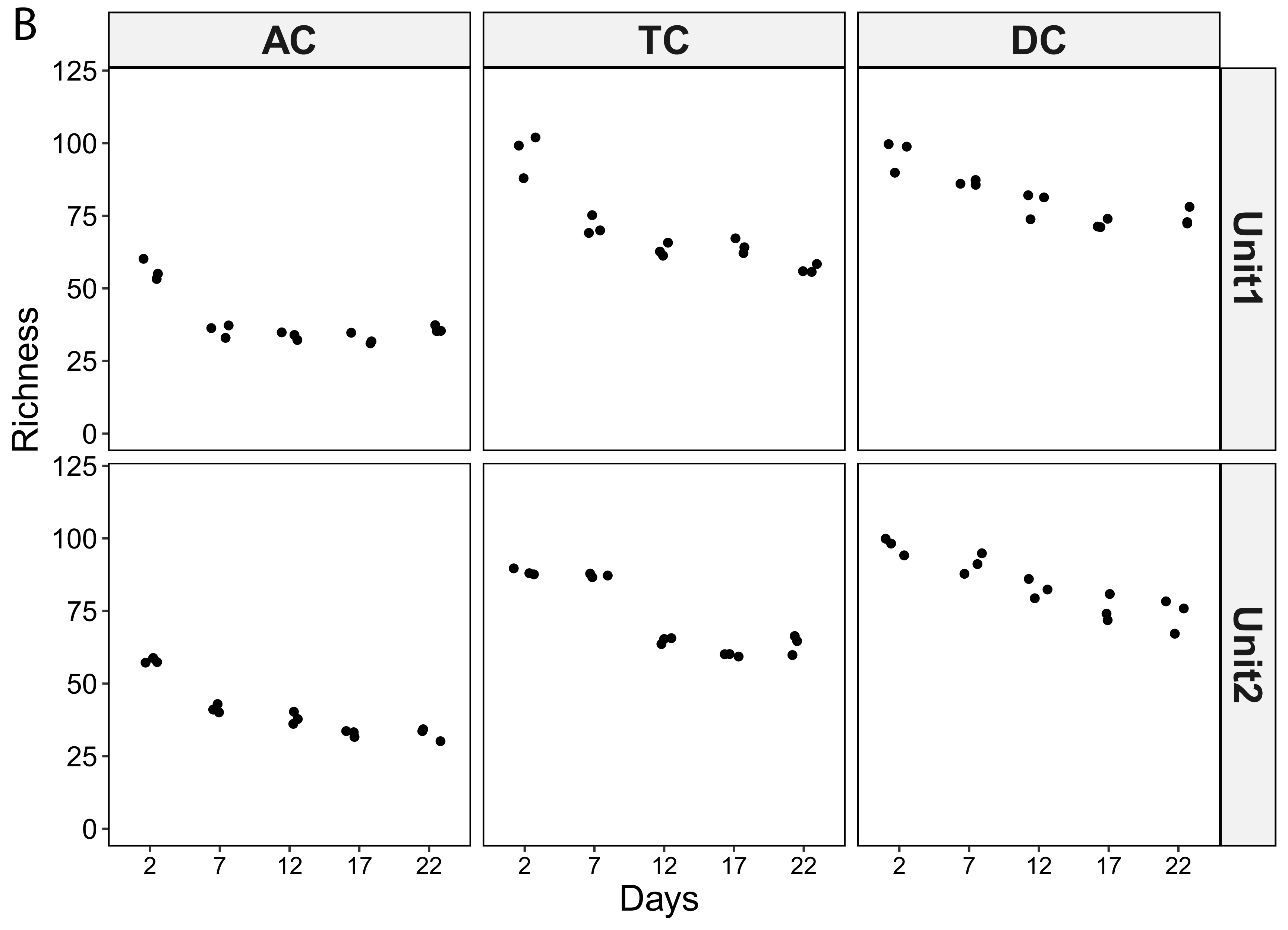

B
AC
TC
DC
Unit1
Unit2
Richness
Days
0
25
50
75
100
125
2
7
12
17
22

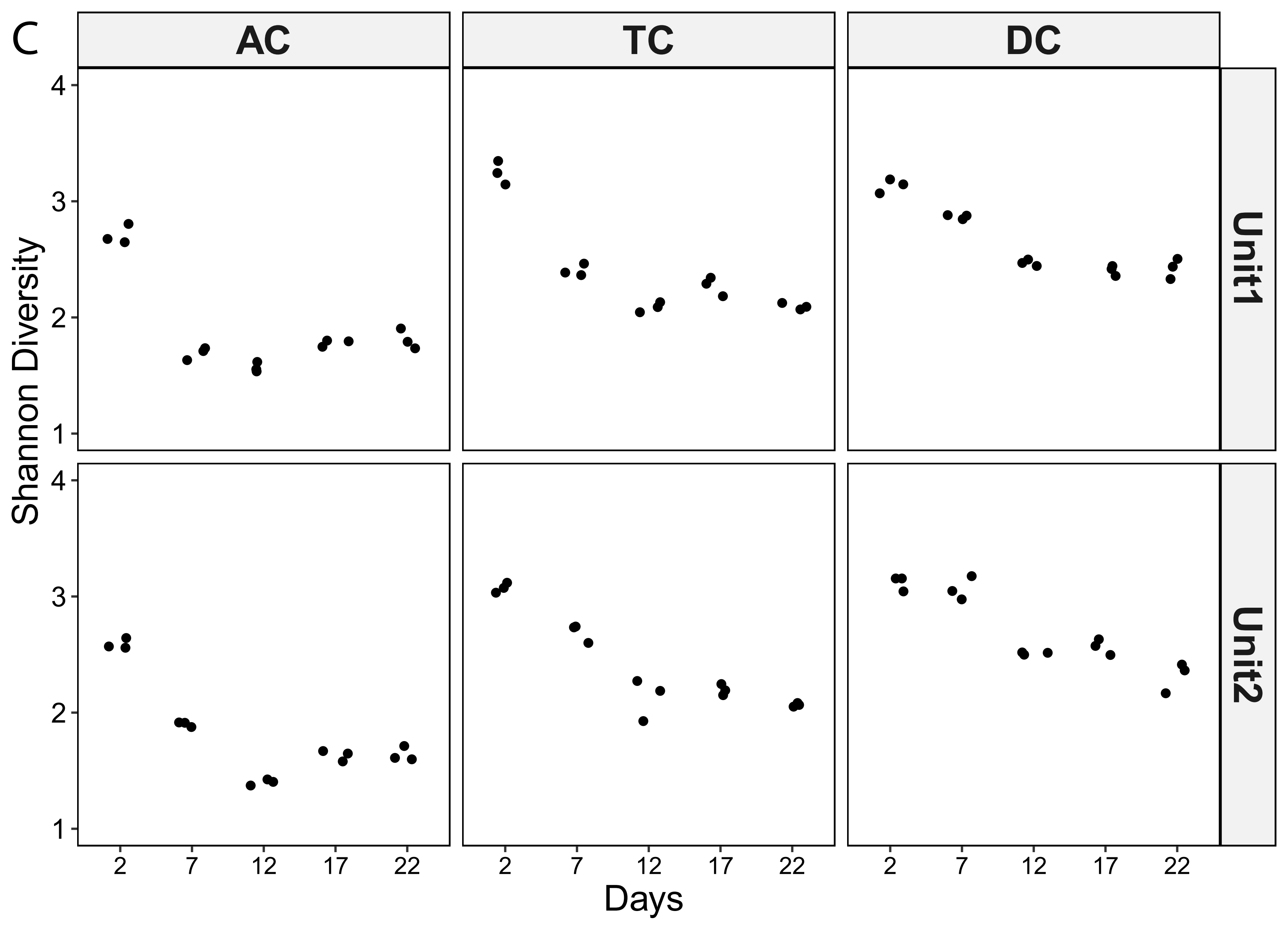
C
AC
TC
DC
Unit1
Unit2
Shannon Diversity
1
2
3
4
2
7
12
17
22
Days

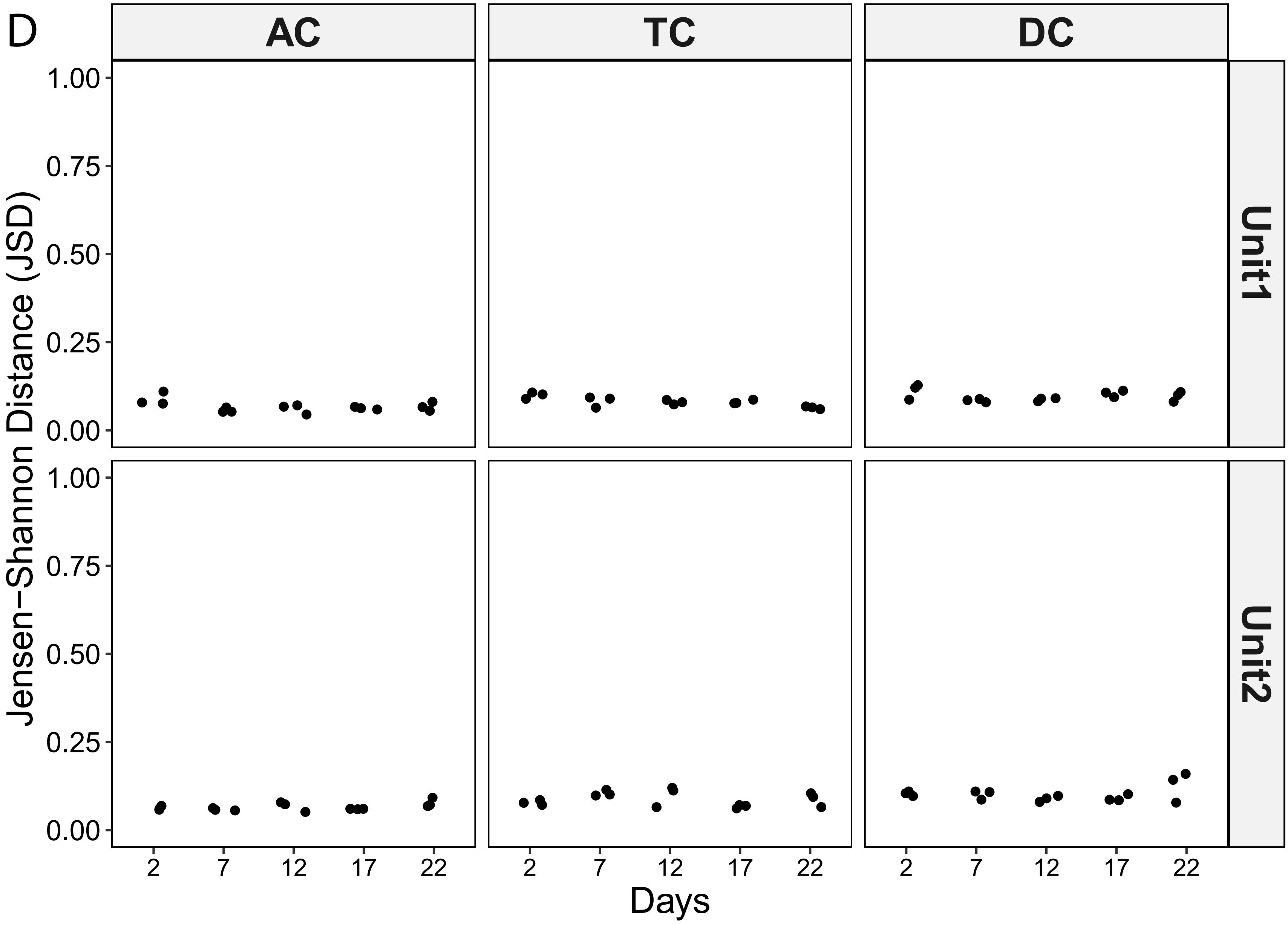
D
AC
TC
DC
Unit1
Unit2
Jensen−Shannon Distance (JSD)
1.00
0.75
0.50
0.25
0.00
2
7
12
17
22
Days
