# Supplementary material for: Ecological Adaptation and Succession of Human Fecal Microbial Communities in an Automated In Vitro Fermentation System
Source: mSystems. 2021 Jul 27;6(4):e00232-21. doi: 10.1128/mSystems.00232-21 (PMC8409738; doi:10.1128/mSystems.00232-21)
Supplement: FIG S6 [file msystems.00232-21-sf006.pdf]

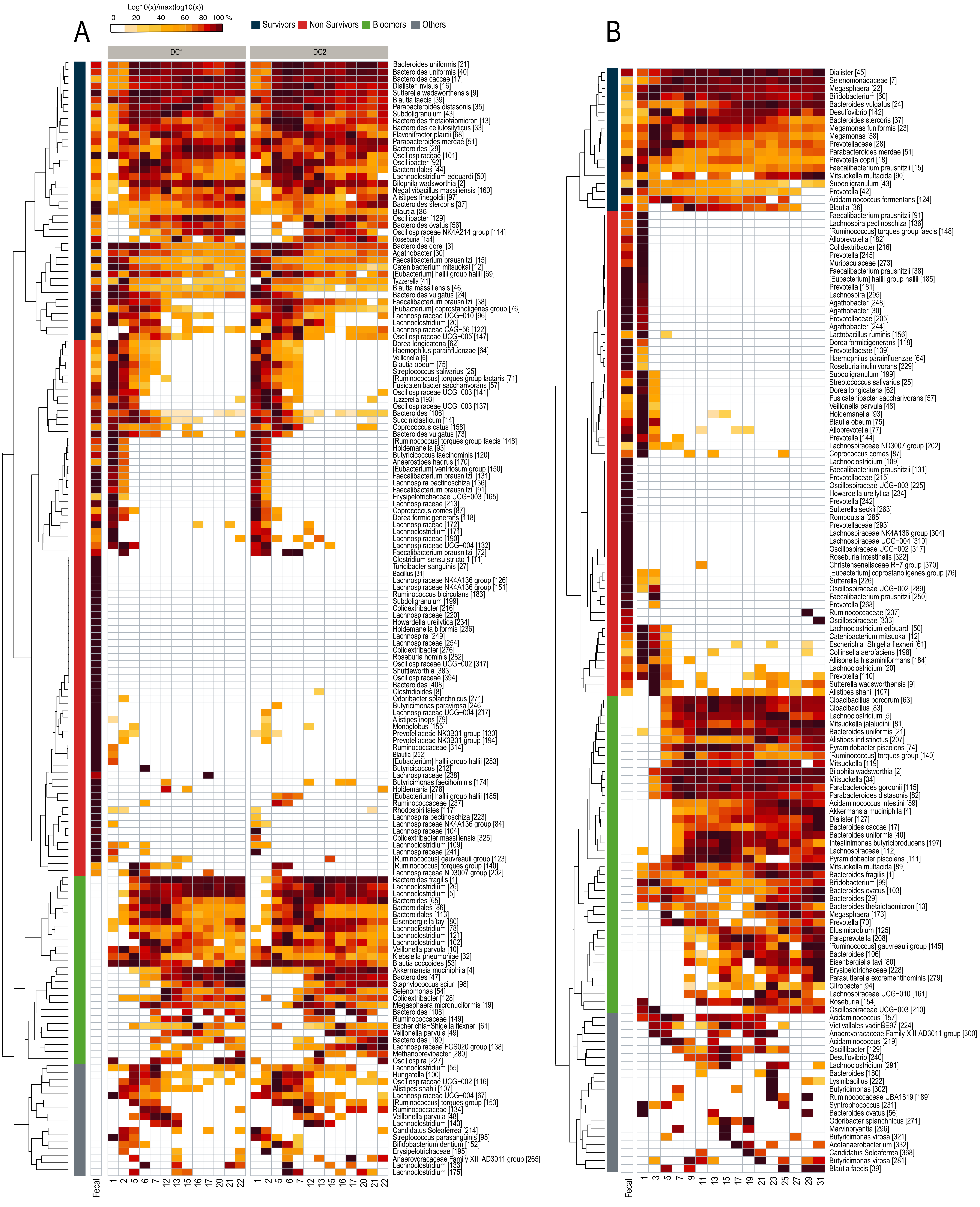
A
B
Log10(x)/max(log10(x))
0 20 40 60 80 100 %
Survivors
Non Survivors
Bloomers
Others
DC1
DC2
Bacteroides uniformis [21]
Bacteroides uniformis [40]
Bacteroides caccae [17]
Dialister invisus [16]
Sutterella wadsworthensis [9]
Blautia faecis [39]
Parabacteroides distasonis [35]
Subdoligranulum [43]
Bacteroides thetaiotaomicron [13]
Bacteroides cellulosilyticus [33]
Flavonifractor plautii [68]
Parabacteroides merdae [51]
Bacteroides [29]
Oscillospiraceae [101]
Oscillibacter [92]
Bacteroidales [44]
Lachnoclostridium edouardi [50]
Bilophila wadsworthia [2]
Negativibacillus massiliensis [160]
Alistipes finegoldii [97]
Bacteroides stercoris [37]
Blautia [36]
Oscillibacter [129]
Bacteroides ovatus [56]
Oscillospiraceae NK4A214 group [114]
Roseburia [154]
Bacteroides dorei [3]
Agathobacter [30]
Faecalibacterium prausnitzii [15]
Catenibacterium mitsuokai [12]
[Eubacterium] hallii group hallii [69]
Tyzzerella [41]
Blautia massiliensis [46]
Bacteroides vulgatus [24]
Faecalibacterium prausnitzii [38]
[Eubacterium] coprostanoligenes group [76]
Lachnospiraceae UCG−010 [96]
Lachnoclostridium [20]
Lachnospiraceae CAG−56 [122]
Oscillospiraceae UCG−005 [147]
Dorea longicatena [62]
Haemophilus parainfluenzae [64]
Veillonella [6]
Blautia obeum [75]
Streptococcus salivarius [25]
[Ruminococcus] torques group lactaris [71]
Fusicatenibacter saccharivorans [57]
Oscillospiraceae UCG−003 [141]
Tuzzerella [193]
Oscillospiraceae UCG−003 [137]
Bacteroides [106]
Succiniclasticum [14]
Coprococcus catus [158]
Bacteroides vulgatus [73]
[Ruminococcus] torques group faecis [148]
Holdemanella [93]
Butyricicoccus faecihominis [120]
Anaerostipes hadrus [170]
[Eubacterium] ventriosum group [150]
Faecalibacterium prausnitzii [131]
Lachnospira pectinoschiza [136]
Faecalibacterium prausnitzii [91]
Erysipelotrichaceae UCG−003 [165]
Lachnospiraceae [213]
Coprococcus comes [87]
Dorea formicigenerans [118]
Lachnospiraceae [172]
Lachnoclostridium [171]
Lachnospiraceae [190]
Lachnospiraceae UCG−004 [132]
Faecalibacterium prausnitzii [72]
Clostridium sensu stricto 1 [11]
Turicibacter sanguinis [27]
Bacillus [31]
Lachnospiraceae NK4A136 group [126]
Lachnospiraceae NK4A136 group [151]
Ruminococcus bicirculans [183]
Subdoligranulum [199]
Colidextribacter [216]
Lachnospiraceae [220]
Howardella ureilytica [234]
Holdemanella biformis [236]
Lachnospira [249]
Lachnospiraceae [254]
Colidextribacter [276]
Roseburia hominis [282]
Oscillospiraceae UCG−002 [317]
Shuttleworthia [383]
Oscillospiraceae [394]
Bacteroides [408]
Clostridioides [8]
Odoribacter splanchnicus [271]
Butyricimonas paravirosa [246]
Lachnospiraceae UCG−004 [217]
Alistipes inops [79]
Monoglobus [155]
Prevotellaceae NK3B31 group [130]
Prevotellaceae NK3B31 group [194]
Ruminococcaceae [314]
Blautia [252]
[Eubacterium] hallii group hallii [253]
Butyricicoccus [212]
Lachnospiraceae [238]
Butyricimonas faecihominis [174]
Holdemania [278]
[Eubacterium] hallii group hallii [185]
Ruminococcaceae [237]
Rhodospirillales [117]
Lachnospira pectinoschiza [223]
Lachnospiraceae NK4A136 group [84]
Lachnospiraceae [104]
Colidextribacter massiliensis [325]
Lachnoclostridium [109]
Lachnospiraceae [241]
[Ruminococcus] gauvreauii group [123]
[Ruminococcus] torques group [140]
Lachnospiraceae ND3007 group [202]
Bacteroides fragilis [1]
Lachnoclostridium [26]
Lachnoclostridium [5]
Bacteroides [65]
Bacteroidales [86]
Bacteroidales [113]
Eisenbergiella tayi [80]
Lachnoclostridium [78]
Lachnoclostridium [121]
Lachnoclostridium [102]
Veillonella parvula [10]
Klebsiella pneumoniae [32]
Blautia coccoides [53]
Akkermansia muciniphila [4]
Bacteroides [47]
Staphylococcus sciuri [98]
Selenomonas [54]
Colidextribacter [128]
Megasphaera micronuciformis [19]
Bacteroides [108]
Ruminococcaceae [149]
Escherichia−Shigella flexneri [61]
Veillonella parvula [49]
Bacteroides [180]
Lachnospiraceae FCS020 group [138]
Methanobrevibacter [280]
Oscillospira [227]
Lachnoclostridium [55]
Hungatella [100]
Oscillospiraceae UCG−002 [116]
Alistipes shahii [107]
Lachnospiraceae UCG−004 [67]
[Ruminococcus] torques group [153]
Ruminococcaceae [134]
Veillonella parvula [48]
Lachnoclostridium [143]
Candidatus Soleaferrea [214]
Streptococcus parasanguinis [95]
Bifidobacterium dentium [152]
Erysipelotrichaceae [195]
Anaerovoracaceae Family XIII AD3011 group [265]
Lachnoclostridium [133]
Lachnoclostridium [175]
Fecal
1 2 5 6 7 12 13 15 16 17 20 21 22
1 2 5 6 7 12 13 15 16 17 20 21 22
Dialister [45]
Selenomonadaceae [7]
Megasphaera [22]
Bifidobacterium [60]
Bacteroides vulgatus [24]
Desulfovibrio [142]
Bacteroides stercoris [37]
Megamonas funiformis [23]
Megamonas [58]
Prevotellaceae [28]
Parabacteroides merdae [51]
Prevotella copri [18]
Faecalibacterium prausnitzii [15]
Mitsuokella multacida [90]
Subdoligranulum [43]
Prevotella [42]
Acidaminococcus fermentans [124]
Blautia [36]
Faecalibacterium prausnitzii [91]
Lachnospira pectinoschiza [136]
[Ruminococcus] torques group faecis [148]
Alloprevotella [182]
Colidextribacter [216]
Prevotella [245]
Muribaculaceae [273]
Faecalibacterium prausnitzii [38]
[Eubacterium] hallii group hallii [185]
Prevotella [181]
Lachnospira [295]
Agathobacter [248]
Agathobacter [30]
Prevotellaceae [205]
Agathobacter [244]
Lactobacillus ruminis [156]
Dorea formicigenerans [118]
Prevotellaceae [139]
Haemophilus parainfluenzae [64]
Roseburia inulinivorans [229]
Subdoligranulum [199]
Streptococcus salivarius [25]
Dorea longicatena [62]
Fusicatenibacter saccharivorans [57]
Veillonella parvula [48]
Holdemanella [93]
Blautia obeum [75]
Alloprevotella [77]
Prevotella [144]
Lachnospiraceae ND3007 group [202]
Coprococcus comes [87]
Lachnoclostridium [109]
Faecalibacterium prausnitzii [131]
Prevotellaceae [215]
Oscillospiraceae UCG−003 [225]
Howardella ureilytica [234]
Prevotella [242]
Sutterella seckii [263]
Romboutsia [285]
Prevotellaceae [293]
Lachnospiraceae NK4A136 group [304]
Lachnospiraceae UCG−004 [310]
Oscillospiraceae UCG−002 [317]
Roseburia intestinalis [322]
Christensenellaceae R−7 group [370]
[Eubacterium] coprostanoligenes group [76]
Sutterella [226]
Oscillospiraceae UCG−002 [289]
Faecalibacterium prausnitzii [250]
Prevotella [268]
Ruminococcaceae [237]
Oscillospiraceae [333]
Lachnoclostridium edouardi [50]
Catenibacterium mitsuokai [12]
Escherichia−Shigella flexneri [61]
Collinsella aerofaciens [198]
Allisonella histaminiformans [184]
Lachnoclostridium [20]
Prevotella [110]
Sutterella wadsworthensis [9]
Alistipes shahii [107]
Cloacibacillus porcorum [63]
Cloacibacillus [83]
Lachnoclostridium [5]
Mitsuokella jalaludinii [81]
Bacteroides uniformis [21]
Alistipes indistinctus [207]
Pyramidobacter piscolens [74]
[Ruminococcus] torques group [140]
Mitsuokella [119]
Bilophila wadsworthia [2]
Mitsuokella [34]
Parabacteroides gordonii [115]
Parabacteroides distasonis [82]
Acidaminococcus intestini [59]
Akkermansia muciniphila [4]
Dialister [127]
Bacteroides caccae [17]
Bacteroides uniformis [40]
Intestinimonas butyriciproducens [197]
Lachnospiraceae [112]
Pyramidobacter piscolens [111]
Mitsuokella multacida [89]
Bacteroides fragilis [1]
Bifidobacterium [99]
Bacteroides ovatus [103]
Bacteroides [29]
Bacteroides thetaiotaomicron [13]
Megasphaera [173]
Prevotella [70]
Elusimicrobium [125]
Paraprevotella [208]
[Ruminococcus] gauvreauii group [145]
Bacteroides [106]
Eisenbergiella tayi [80]
Erysipelotrichaceae [228]
Parasutterella excrementihominis [279]
Citrobacter [94]
Lachnospiraceae UCG−010 [161]
Roseburia [154]
Oscillospiraceae UCG−003 [210]
Acidaminococcus [157]
Victivallales vadinBE97 [224]
Anaerovoracaceae Family XIII AD3011 group [300]
Acidaminococcus [219]
Oscillibacter [129]
Desulfovibrio [240]
Lachnoclostridium [291]
Bacteroides [180]
Lysinibacillus [222]
Butyricimonas [302]
Ruminococcaceae UBA1819 [189]
Syntrophococcus [231]
Bacteroides ovatus [56]
Odoribacter splanchnicus [271]
Marvinbryantia [296]
Butyricimonas virosa [321]
Acetanaerobacterium [332]
Candidatus Soleaferrea [368]
Butyricimonas virosa [281]
Blautia faecis [39]
Fecal
1 3 5 7 9 11 13 15 17 19 21 23 25 27 29 31
